# Atomic structure and formation mechanism of a newly discovered charge density wave in the *m* = 2 monophosphate tungsten bronze

**DOI:** 10.1107/S2052252526002836

**Published:** 2026-05-29

**Authors:** Arianna Minelli, Elen Duverger-Nedellec, Olivier Perez, Alain Pautrat, Adrien Girard, Johnathan Bulled, Marek Mihalkovič, Marc de Boissieu, Alexei Bosak

**Affiliations:** a European Synchrotron Radiation Facility BP 220 F-38043Grenoble Cedex France; b Department of Chemistry University of Oxford South Parks Road OxfordOX1 3QR United Kingdom; c Neutron Scattering Division Oak Ridge National Laboratory,Oak Ridge TN 37831 USA; d University of Bordeaux CNRS Bordeaux INP ICMCB UMR 5026 F-33600Pessac France; e CRISMAT-ENSICAEN University of Caen Basse-Normandie CNRS/UMR 6508 6 Bd Maréchal Juin 14050 Caen Cedex 4 France; f Sorbonne Université CNRS MONARIS F-75252Paris France; g Institute of Physics Slovak Academy of Sciences Dúbravskà cesta 9 Bratislava84511 Slovak Republic; h University of Grenoble Alpes CNRS SIMAP 38000Grenoble France; IRCP Chimie-ParisTech, France

**Keywords:** correlated fluctuations, dynamical studies, monophosphate tungsten bronzes, charge density waves, 1D conductors

## Abstract

The *m* = 2 member of the monophosphate tungsten bronze family had been thought to be the only one without an electronic instability at low temperature. In this paper, we report the discovery of a charge density wave phase in this compound, with a transition temperature of 290 K and an incommensurate modulation vector **q** = 0.245b* + ξc*, which reaches a lock-in with a commensurate vector at 130 K. The presence of this new phase is confirmed by diffraction and resistivity measurements. Pre-transitional dynamics are investigated using diffuse and inelastic X-ray scattering, revealing a clear Kohn anomaly. We analyze both structural and electronic contributions to the phase transition, providing a comprehensive picture of the mechanism driving this newly identified instability.

## Abstract 2

A charge density wave (CDW) instability is discovered in the *m* = 2 monophosphate tungsten bronze, with an incommensurate modulation **q** = 0.245b* + 0.02c* appearing at 290 K and locking in to the commensurate vector **q** = 0.25b* at 130 K. Diffraction, resistivity and diffuse/inelastic X-ray scattering jointly establish the transition mechanism, showing that lattice distortions dominate while the electronic CDW is only weakly coupled to the structural reorganization.

## 1. Introduction

Low-dimensional metals exhibit peculiar electronic properties, such as superconductivity and charge or spin density waves (CDWs, SDWs). The charge density wave (CDW) phase, where electrons condense in momentum space, was first linked to an instability described by Peierls in 1955 (Peierls, 1955 ▸). Peierls introduced the intrinsic instability in a one-dimensional (1D) metallic chain as a mathematical concept, in which the parabolic electronic band of non-interacting particles – subjected to a periodic external potential – has a Fermi wavevector determined by the chemical potential. For a half-filled band, the system becomes unstable with respect to a doubling of the unit cell and toward a periodic lattice distortion, which leads to the opening of an energy gap. This type of instability has been observed in many real materials that exhibit quasi-1D behavior and linear-chain correlations (Peierls, 1991 ▸). Building on this concept, CDWs have been classified in various ways (Rossnagel, 2011 ▸; Zhu *et al.*, 2015 ▸; Pouget & Canadell, 2024 ▸) and are commonly classified according to the strength of electron–phonon coupling, which defines two limiting regimes (Pouget & Canadell, 2024 ▸; Motizuki, 1986 ▸; Radić, 2025 ▸; Aubry *et al.*, 1992 ▸; Aubry & Quemerais, 1989 ▸): (i) weak-coupling CDWs, where lattice distortion and electronic modulation are only weakly coupled as in the Peierls model, and (ii) strong-coupling CDWs, characterized by larger lattice distortions, wider energy gaps and modified Kohn anomaly behavior.

In studies of CDW phase transitions, structural and electronic effects are typically considered as arising from the same instability, rather than being treated as distinct contributions. Real structures, even in low-dimensional metals, deviate significantly from the idealized one-dimensional metallic chain. In quasi-1D systems, deviations from simple Peierls-type theory must be considered. As Johannes and Mazin, and later Pouget and Canadell, have argued (Johannes & Mazin, 2008 ▸; Pouget & Canadell, 2024 ▸), it is not possible to fully describe the complexity of CDW transitions using only the Peierls mechanism and the concept of perfect Fermi surface nesting.

**Figure 1:**
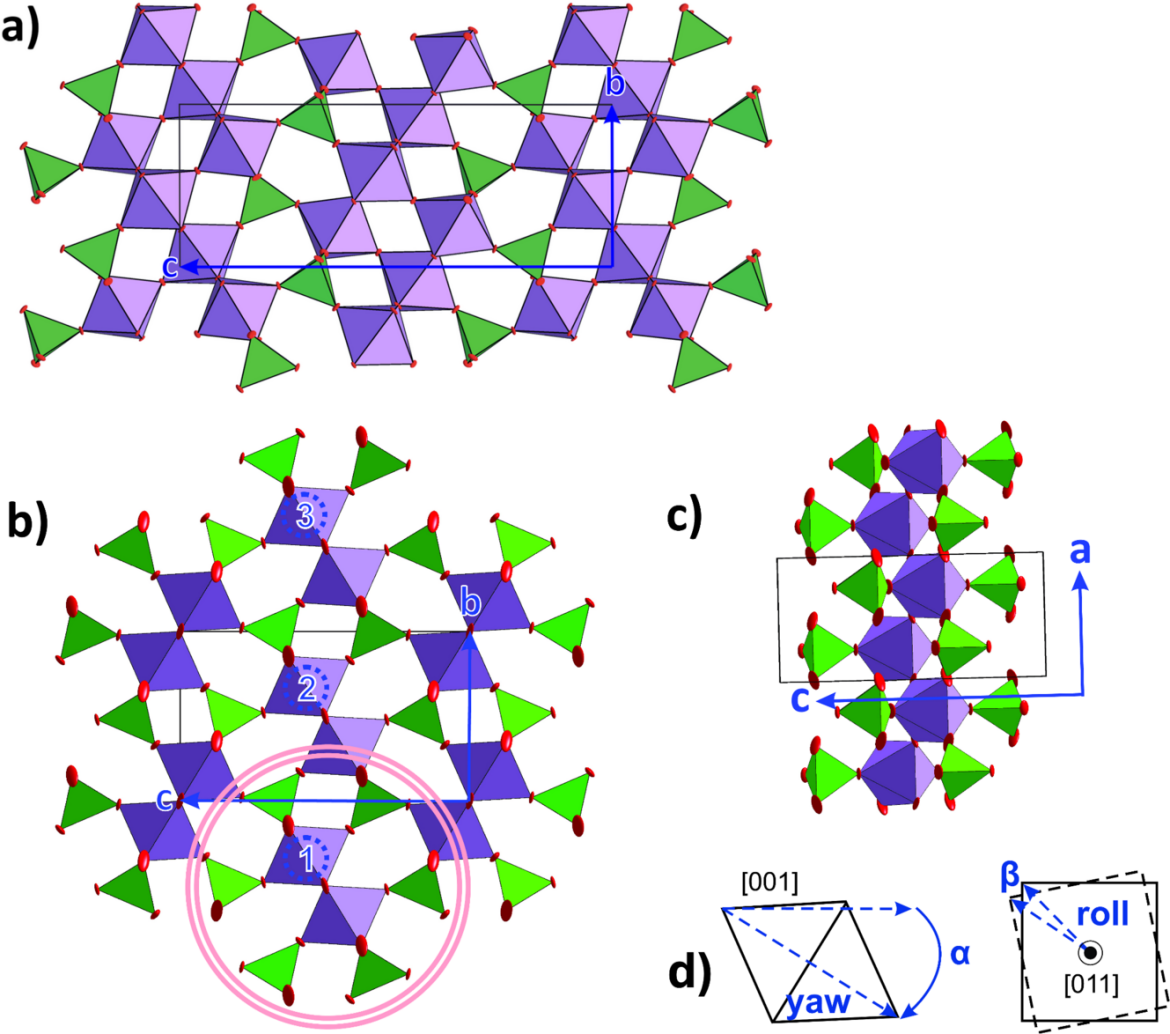
(*a*) Projection along **a** of the structure of the *m* = 4 member P_4_W_8_O_32_ showing the typical structure of the monophosphate tungsten bronze family. (*b*) Structure of P_4_W_4_O_20_ at room temperature: projection along **a**. The elementary motif is highlighted with a double pink circle. The chains of first neighbors are drawn beyond this pink circle using the same dark purple color as the elementary chain. (*c*) View of the (WO_6_)_∞_ chains running along **a**. (*d*) The description of the yaw, on the left, and roll axes, on the right. These axes have libration angles α and β, which describe the rigid-body motions of the octahedra. These oscillations are described in Section 6.2.

To highlight the need for a detailed analysis that separates structural and electronic contributions to the phase transition and thus their contributions to the instability that generates the CDW phase, we studied a relatively simple oxide with a quasi-1D electronic structure. The lowest member of the monophosphate tungsten bronze family, (PO_2_)_4_(WO_3_)_2*m*_, consists of zigzag chains of WO_6_ octahedra surrounded by PO_4_ tetrahedra [see Fig. 1 ▸(*b*) and (*c*)]. Other members of this family show an ReO_3_-type layered structure composed of tungsten-oxide slabs and phosphate tetrahedra monolayers [see Fig. 1 ▸(*a*)], leading to a quasi-2D instability. A comprehensive overview of this family of compounds, encompassing both their structural and electronic properties, can be found in a number of articles, monographs and review papers (*e.g.* Foury *et al.*, 1993 ▸; Foury & Pouget, 1993 ▸; Ottolenghi *et al.*, 1995 ▸; Greenblatt, 1996 ▸; Schlenker, 1996 ▸; Ottolenghi & Pouget, 1996 ▸; Foury *et al.*, 1999 ▸; Roussel *et al.*, 2001 ▸; Pérez *et al.*, 2013 ▸).

Our combined structural, electronic and dynamical studies on this compound provide a comprehensive picture of the phase transition, allowing us to disentangle the contributions from the lattice and electronic subsystems. Moreover, our results show that the structural instability is not entirely driven by the electronic CDW. This analysis is made possible through diffraction, diffuse scattering, physical measurements, inelastic X-ray scattering and molecular dynamics simulations.

## 2. Previous results

There are few papers concerning the lowest member of the monophosphate tungsten bronzes family, *m* = 2, probably because no phase transition had been observed by electrical resistivity measurements and so it was considered unimportant (Teweldemedhin *et al.*, 1991 ▸). However, there is a quasi-one-dimensional character along the direction of the zigzag chain of W octahedra. The resistivity is about one to two orders of magnitude lower than along the other directions (Teweldemedhin *et al.*, 1991 ▸). A paper by Canadell and Whangbo presents a tight-binding model that predicts a possible electronic instability along the direction of the chain, which is due to the localized and delocalized electrons (Canadell & Whangbo, 1990 ▸).

The synthesis of two different forms of PWO_5_ is reported by Kinomura *et al.* (1988 ▸). The first form, obtained *via* high-pressure synthesis (using 6 GPa), exhibits a tetragonal unit cell with *a* = 6.25 Å and *c* = 4.07 Å, making it isostructural to MoPO_5_. The second form is prepared following the classical protocol used for monophosphate tungsten bronzes (MPTBs). It has a pseudo-orthorhombic cell with *a* = 11.172 (8) Å, *b* = 5.217 (1) Å, *c* = 6.543 (2) Å, α = 90°, β = 90.34 (4)° and γ = 90° (Kinomura *et al.*, 1988 ▸). The structure analysis of this latter form is reported at room temperature by Wang *et al.* as the second member of the monophosphate tungsten bronzes with pentagonal tunnels (MPTBp) family (Wang *et al.*, 1989 ▸) and it is the member presented in this paper. Despite the angle β differing slightly from 90°, the authors claimed it had ortho­rhombic symmetry.^1^ In the paper by Wang *et al.*, the noncentrosymmetric space group *Pna*2_1_ is considered for the structure solution (Wang *et al.*, 1989 ▸) on the basis of the systematic absences of reflections and statistical tests. The authors reported ‘only the W atom was refined with anisotropic temperature factors since most of the light atoms gave non-positive definite values’. They also noted high atomic displacement parameter (ADP) values for three out of five oxygen atoms. The final agreement factor for their study was *R* = 0.0273 for 547 reflections with *I* ≥ 3σ(*I*). However, the slight deviation of the β angle from 90° as well as the difficulty in refining anisotropic ADPs require some clarification, especially since an accurate analysis of the structural state observed below the CDW phase transition has to be performed. The present structural analysis attempts both to provide additional information on the actual symmetry at room temperature and to resolve the structure at low temperatures.

## 3. Synthesis

The method used for synthesis and crystal growth is described by Roussel *et al.* (1996 ▸). Using a mixture of (NH_4_)_2_HPO_4_, WO_3_ and W precursors in a stoichiometric ratio normally leads to the formation of tungsten diphosphate materials. However, an alternative source of phosphorus, P_2_O_5_, is required to successfully synthesize the *m* = 2 member. This final mixture must be prepared in a glove box and later put in an evacuated, sealed quartz tube. The ampoule is introduced into a furnace with a temperature gradient.

Crystals of MPTBp with *m* = 2 with a truncated parallelepipedic shape and up to millimetres in size were isolated. The largest ones were used for measurements of physical properties and the smaller crystals were used for X-ray diffraction, diffuse scattering and inelastic scattering experiments.

## 4. Experimental methods

The quality of the crystals was checked by diffraction in the laboratory and with synchrotron radiation at the European Synchrotron Radiation Facility (ESRF).^2^ Diffraction data collection was performed using a Synergy-S Rigaku diffractometer with an Mo microfocus source. A series of measurements for the thermo-diffraction experiment were taken from 290 K to 80 K using the same strategy (same sample-to-detector distances, same current characteristics for the Mo X-ray sources, same exposure time and same value of λ/2(sin θ_max_) = 0.8 Å [*i.e* θ_max_ = 26.315° or sin(θ_max_)/λ = 0.624 Å^−1^]. The temperature ramp was set to 1° per minute and 10 minutes of stabilization was imposed before each measurement. For each data collection, *Crysalis^pro^* (Agilent, 2014 ▸) was used both for reconstructing oriented diffraction planes from experimental frames and integrating the data.

The diffuse scattering (DS) and inelastic X-ray scattering (IXS) experiments were conducted on the ID28 beamline at the ESRF. A bar-shaped sample 0.15 × 0.8 × 0.2 mm in size was used for temperature-dependence measurements, which were performed with a Cryostream (limited to ∼90 K). The sample was oriented with the *a* axis as the rotation axis perpendicular to the beam for the IXS measurement. A monochromatic beam of λ = 0.6968 Å, corresponding to an energy of 17.79 keV, was used for the DS measurements, whereas the high-energy-resolution setup was required for IXS. The latter used a beam of 23.72 keV to reach an energy resolution of 1.5 meV.

For the DS measurements, the sample was rotated through 360° orthogonal to the incoming beam, oriented at 45° with respect to the horizontal plane. Single frames were collected with an angular slicing of 0.1°. A Pilatus 1M (Dectris) detector with a pixel size of 172 × 172 µm was used in single-photon-counting mode (Dectris, 2013 ▸). The *Crysalis^pro^* software package was used to obtain the orientation matrix and to perform a preliminary data evaluation for the subsequent IXS measurements (Agilent, 2014 ▸). The diffuse scattering maps were created using software developed locally for ID28 called *Project X*. Further details about the experimental setup for DS and IXS at ID28 have been reported elsewhere (Girard *et al.*, 2019 ▸; Krisch & Sette, 2007 ▸).

For resistivity measurements, four gold pads were evaporated onto a single crystal of size 0.80 × 0.45 × 0.40 mm. Gold wires were then attached using silver epoxy Dupont 6828. All measurements were performed using a PPMS (Quantum Design), using either the resistivity option or an external low noise current source (ADRET 103A) and voltmeter (Keithley 2182A) for *V*(*I*) curves, and a home-made amplifier/dynamic acquisition card setup for noise measurements (Scola *et al.*, 2005 ▸).

## 5. Molecular dynamics simulations

*Ab initio* molecular dynamics (MD) simulations were performed using density functional theory as implemented in the plane-wave code *VASP* (Kresse & Furthmuller, 1996 ▸). The computational setup employed a 5 × 1 × 3 **k**-point grid and projector augmented wave (PAW) potentials (Kresse & Joubert, 1999 ▸). The exchange–correlation energy was described using the Perdew–Burke–Ernzerhof generalized gradient approximation (PBE-GGA), and spin polarization was included in all calculations.

Simulations were carried out in the canonical (NVT) ensemble with a constant volume. Temperature control was achieved via velocity rescaling every 10 steps. The system was equilibrated over 1500 steps using a time step of 3 fs. Production runs were subsequently performed for 9000 steps with a time step of 5 fs.

## 6. Results

We present our results as follows. In Section 6.1, we discuss the structural results obtained from diffraction measurements performed on both the high-symmetry (HS) phase and the CDW phase. In Section 6.2, MD simulations and symmetry analysis provide insight into the instabilities present in the HS phase, which are linked to the low-temperature CDW phase. Section 6.3 focuses on the pre-transitional phonon instability, investigated using DS and IXS. Finally, in Section 6.4, we examine the electronic transport properties through resistivity measurements, revealing their connection to the CDW phase transition.

### 6.1. Structural analysis

#### 6.1.1. Crystal structure in the fundamental state

A first series of crystals were selected from our own batch synthesis; the samples that were chosen had a parallelepipedic shape and a size of about 100 × 100 × 100 µm. Data collections were performed using a Synergy-S Rigaku diffractometer with an Mo microfocus source. Analysis of the reciprocal space led to the unit cell *a* = 5.2368 (2) Å, *b* = 6.5683 (2) Å, *c* = 11.2108 (3) Å, α = 90.217 (2)°, β = 90.000 (2)°, γ = 90.001 (2)° and *V* = 385.614 (12) Å^3^; the setting chosen here is the one used for all members of the MPTBp family. As previously reported, a significant deviation of the α angle [the β angle in the setting used by Wang *et al.* (1989 ▸)] from 90° was found. Moreover, additional weak reflections with *h* = ½ and *l* = ½ were observed. The introduction of three twin domains related by a threefold axis parallel to the *b* axis allowed these reflections to be indexed.

To eliminate any doubts about the quality of the crystals and in particular the influence of twinning on both the metrics and the refinements, a new crystal of smaller dimensions (14 × 41 × 62 µm) but showing sharp Bragg reflections and at first glance the absence of twinning was selected. Data collection was performed at room temperature (RT), also using the Synergy-S Rigaku diffractometer with the Mo microfocus source and an Eiger 1M Dectris photon-counting detector, and led to the following unit cell: *a* = 5.22786 (5) Å, *b* = 6.55814 (11) Å, *c* = 11.1883 (2) Å, α = 90.0583 (15)°, β = 90.0118 (12)°, γ = 90.0024 (10)°, *V* = 383.591 (10) Å^3^. The deviation of the α angle from 90° was much smaller for this sample.^3^ Although it remains difficult to completely rule out a possible lowering of symmetry toward a monoclinic space group, the overall observations, together with the data integration done using the *mmm* Laue class, leading to *R*_int_ = 0.05, are in favor of an orthorhombic symmetry. The analysis of the diffraction pattern shows the following conditions limiting the diffraction: 0*k*0: *k* = 2*n*; 00*l*: *l* = 2*n*; *h*0*l*: *l* = 2*n*; *h*00: *h* = 2*n*; *hk*0: *h* + *k* = 2*n*, leading to two possible space groups: *Pmcn* or *P*2_1_*cn*. The monoclinic model that was considered is reported in Appendix 1 in the supporting information and the results obtained do not support the monoclinic description.

Reflections statistics (a Wilson plot leads to 〈|*E*^2^ − 1|〉 = 0.942) were in favor of the centrosymmetric space group. However, since the results of the literature were in favor of the noncentrosymmetric space group, two concurrent *ab initio* structure solutions were performed, one in each space group. In both cases, following data integration using *Crysalis^pro^* (Agilent, 2014 ▸), the structure was solved using *Superflip* (Oszlányi & Sütő, 2004 ▸) and the charge-flipping algorithm (Palatinus & Chapuis, 2007 ▸), and refinement was performed with *Jana2006* (Petříček *et al.*, 2014 ▸).

The structural analysis of P_4_W_4_O_20_ with the centrosymmetric space group required the introduction of one tungsten, one phosphorus and four oxygen atoms. The final agreement factor was *R*_obs_ = 2.08% for 952 independent reflections with *I* ≥ 3σ(*I*) and 41 refined parameters; the ADPs of all atoms considered as anisotropic were positive definite.

In the noncentrosymmetric case, the structure is fully described using one tungsten, one phosphorus and five oxygen atoms. The final agreement factor was *R*_obs_ = 2.02% for 1700 independent reflections with *I* ≥ 3σ(*I*) and 65 refined parameters, but the anisotropic ADP of one oxygen atom was not positive definite. Moreover, two twin domains related by an inversion center had been introduced and the refined twin volumes were equal to 0.50 (4)/0.50 (4).

In both cases, the ADPs show elongated displacement ellipsoids along **a** and **b**, in particular for the oxygen atoms. The absence of an inversion center does not eliminate this anomaly. The atomic parameters corresponding to the two options are proposed as supplementary materials (Appendices 1–3). All these different features are in agreement with the centrosymmetric hypothesis.

The structure will be thus be described using the results obtained using orthorhombic symmetry and the centrosymmetric space group. Fig. 1 ▸(*b*) shows the building principle of the *m* = 2 MPTBp; a pink double circle in Fig. 1 ▸(*b*) shows the elementary motif. When viewing this elementary building block projected along **b** [Fig. 1 ▸(*c*)], a zigzag chain of edge-sharing WO_6_ octahedra (with six W—O distances range from 1.84 to 1.97 Å) running along **a** can be identified. Each octahedron is connected to four PO_4_ tetrahedra (with four P—O distances range from 1.497 to 1.516 Å). Consequently, the (WO_6_)_∞_ chains observed in Fig. 1 ▸(*b*) and (*c*) are isolated from each other, revealing the 1D character of the structure of P_4_W_4_O_20_. We note that the analysis of the ADPs shows elongated ellipsoids along **a** and **b** for the oxygen atoms.

#### 6.1.2. Thermo-diffraction screening

**Figure 2:**
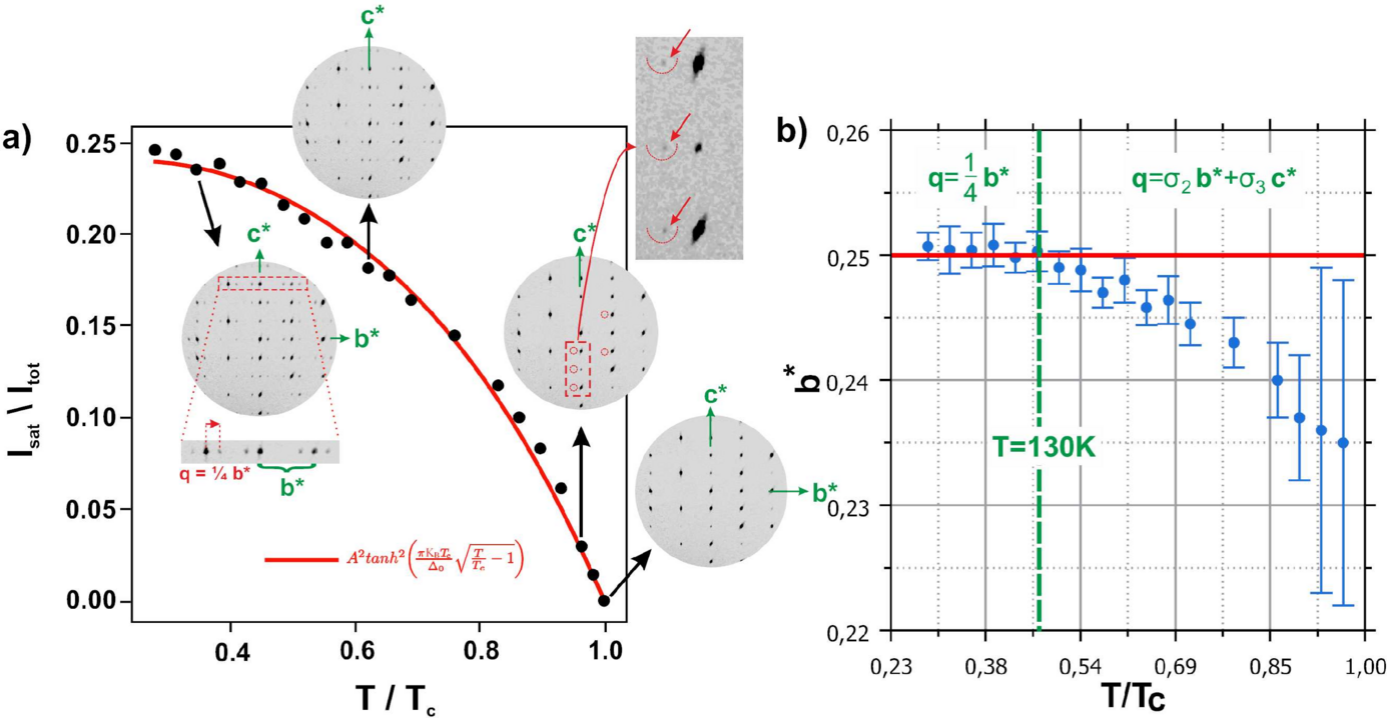
(*a*) Evolution of the sum of the intensities of all the observed satellite reflections versus the sum of the intensities of all reflections with *I* ≥ 3σ(*I*), as a function of the normalized temperature (*T*_c_ = 290 K). The same area of the (0*KL*) plane is plotted for different temperatures. Below *T*_c_, weak satellite reflections are observed (red arrows). The solid line is a fit given by the (squared) expression of the BCS gap in the weak coupling limit (Δ_0_/*KT*_c_ = 1.76), see the text. (*b*) Evolution of the component of **q** along b* versus *T*.

The single crystal selected for structural analysis at room temperature, chosen for its absence of twinning, was subsequently used for the laboratory thermo-diffraction experiments. A series of measurements were carried out between 290 K and 80 K using an identical experimental strategy throughout: the same crystal, the same detector distance, identical Mo X-ray source operating conditions, the same exposure time, and a constant resolution limit defined by λ × 2 sin(θ_max_) = 0.8 Å [corresponding to θ_max_ = 26.315° or sin(θ_max_)/λ = 0.624 Å^−1^]. The temperature was varied at a rate of 1 K per minute, and a stabilization time of 10 minutes was allowed before each data collection. In total, 21 complete datasets were recorded. At each temperature *Crysalis^pro^* (Agilent, 2014 ▸) was used to reconstruct oriented diffraction planes from the experimental frames and to perform data integration. Selected regions of the (0*KL*) plane are shown in Fig. 2 ▸. Starting at approximately 280 K, additional reflections appear at positions close to (1/4)b*. To determine the components of the modulation vector **q** without imposing symmetry constraints, all datasets were integrated assuming triclinic symmetry. No significant components were detected along a* or c*. However, as shown in Fig. 2 ▸(*b*), the component along b* exhibits a clear deviation from the commensurate value of 1/4. The same figure indicates a lock-in phenomenon occurring near 130 K, where the b* component stabilizes at exactly 1/4. These results indicate that the *m* = 2 compound adopts an incommensurate structure immediately below the phase transition and remains incommensurate down to approximately 130 K, where a transition to a commensurate state occurs. The intensity of the satellite reflections [Fig. 2 ▸(*a*)] is very weak close to the transition temperature but increases rapidly upon cooling, reaching a near-plateau around 80 K. At this temperature, the satellites account for approximately one-quarter of the total diffracted intensity, as illustrated in Fig. 2 ▸.

**Figure 3:**
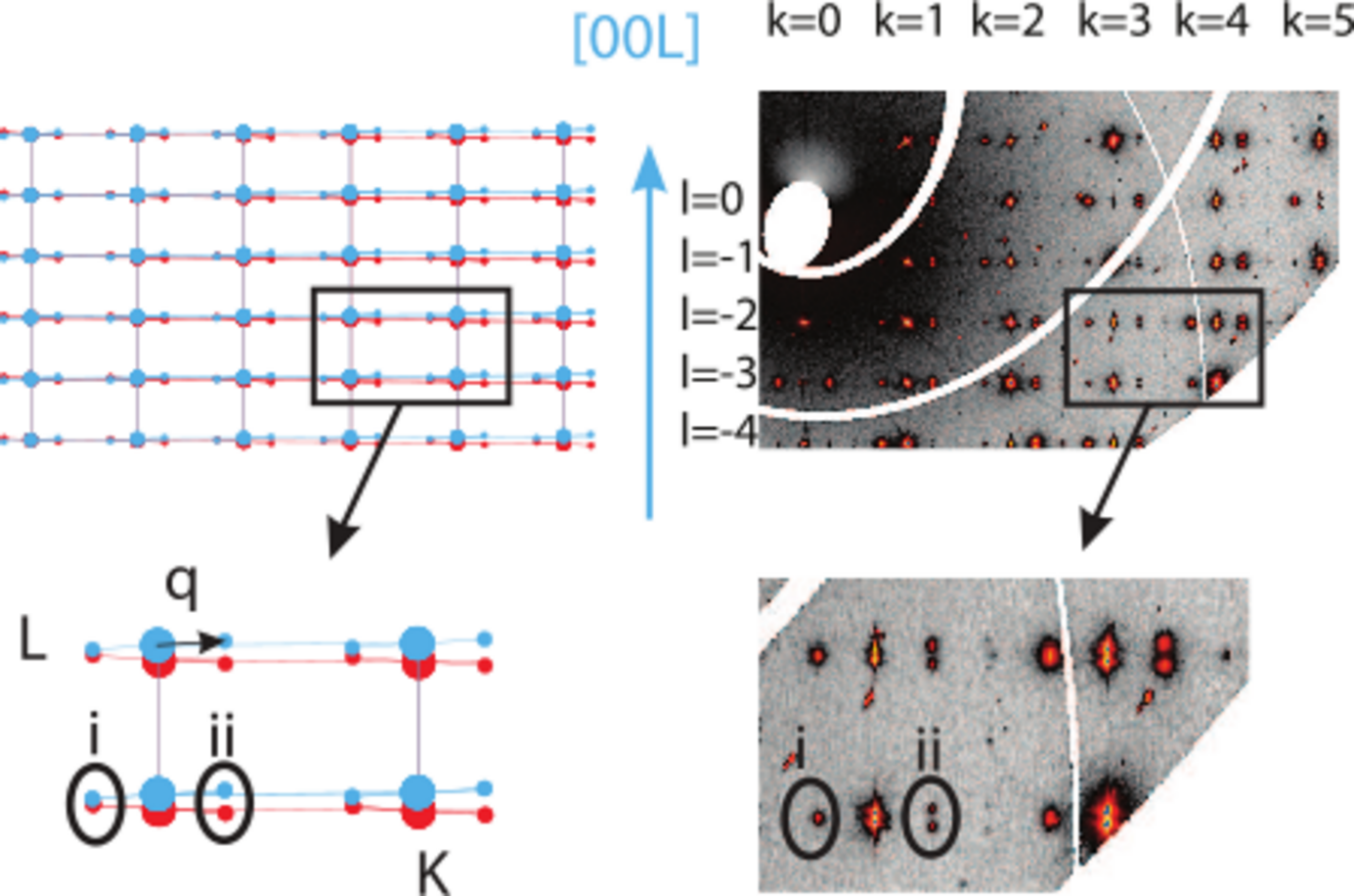
Right: (0*KL*) plane measured at the ESRF for DS in the CDW state at 155 K. The magnified areas i and ii show the absence of splitting of the −1 satellite and the splitting of the +1 satellite, respectively, observed for high *K*. Left: model for the (0*KL*) plane built with **q** = 0.25b* − 0.019c*, the unit cell *a* = 5.223 Å, *b* = 6.548 Å, *c* = 11.191 Å, α = 89.6°, β = γ = 90°, and two domains (blue and red) related by a twofold axis parallel to **b**.

High-*Q*-resolution diffuse scattering measurements were subsequently performed at the ESRF over a broad temperature range spanning both sides of the phase transition (Fig. S2). It is important to note that P_4_W_4_O_20_ absorbs X-rays strongly, leading to significant beam-induced heating. The crystal temperature could rise by up to approximately 100°C under the beam, introducing uncertainty in the absolute temperature determination and preventing measurements at the lock-in temperature. Despite these limitations, the ESRF data revealed several key features that provide insight into the symmetry of both the ground state and the CDW phase. Below the transition, well defined satellite reflections develop at close to 0.25b*. Because the main Bragg reflections are saturated over the entire temperature range, the analysis focuses on the satellite reflections, which carry the relevant signatures of the phase transition. The elastic satellite signals exhibit a weak but systematic broadening along c*. This anisotropic broadening is consistent with a slight monoclinic distortion and suggests the presence of twin domains already in the low-temperature phase. The effect becomes more pronounced upon cooling (see Fig. S2). Fig. 3 ▸ shows data collected below the transition at approximately 160 K. In the (0*KL*) diffraction plane (the right-hand side of Fig. 3 ▸), both main Bragg reflections and satellite reflections are clearly visible. As *k* increases, the main reflections display anisotropic broadening along c*. Even more strikingly, the +1-order satellite reflections exhibit an increasing splitting with increasing *k*, whereas the −1-order satellites do not show comparable splitting. This asymmetric behavior strongly indicates a twinned domain structure, most likely resulting from a lowering of symmetry towards a monoclinic Laue class. Quantitative analysis of the main reflection broadening yields a monoclinic angle α ≃ 89.6°. However, neither this small angular deviation nor a twin relationship involving a 180° rotation around **b** or **c** is sufficient to reproduce the observed asymmetry of the satellite reflections (notably those labeled i and ii in Fig. 3 ▸, right panel). To account for these features, it is necessary to introduce an additional component along c* in the modulation vector. The best agreement with the experimental diffraction pattern is obtained with **q** = 0.25b* + 0.019c*; Fig. S3 clarifys the effect of this additional component. Simulations performed using lattice parameters *a* = 5.223 Å, *b* = 6.548 Å, *c* = 11.191 Å, α = 89.6°, β = γ = 90° and two domains related by a 180° rotation about **a** reproduce the experimental (0*KL*) plane remarkably well (Fig. 3 ▸, left panel), including the characteristic behavior of satellites i and ii. This two-component modulation vector is incompatible with the orthorhombic *mmm* Laue class and therefore strongly supports a monoclinic symmetry for the modulated phase. Moreover, the non-zero c* component confirms the incommensurate character of the modulation at 160 K. P_4_W_4_O_20_ thus appears to adopt an incommensurate monoclinic modulated structure from the phase-transition temperature down to at least 160 K. Experimental constraints prevented further investigation at lower temperatures under high-resolution conditions.

In contrast, the laboratory single-crystal X-ray diffraction data collected between room temperature and 80 K do not provide direct evidence of symmetry lowering. The weak monoclinic distortion and the small c* component of the modulation vector remain below the resolution limit of the in-house experiment. Nevertheless, these measurements clearly reveal the lock-in transition near 150 K, where the modulation vector converges to **q** = 0.25b*, consistent with a commensurate modulation below this temperature. The structural refinement at 80 K presented in the previous section, in particular the *t*_0_ section of the superspace description leading to the supercell space group *P*2_1_/*m*11, is fully consistent with both the slight deviation of α from 90° and the commensurate character of the low-temperature modulation.

The structural models proposed above and below the phase transition therefore provide a reliable approximation of the structure of MPTBp (*m* = 2) in both the ground state and the CDW state. Furthermore, this structural description yields improved agreement with molecular dynamics simulations. It is this physically consistent approximation of the structure that is used in the following section for further analysis.

#### 6.1.3. Crystal structure in the CDW state: analysis at 80 K

Following the thermo-diffraction investigation and to obtain maximum intensity of the satellite reflections, a full data collection was performed at 80 K up to λ/(2 sin θ_max_) = 0.6 Å [*i.e.* θ_max_ = 36.234° or sin(θ_max_)/λ = 0.832 Å^−1^]; as for all the thermo-diffraction experiments, the data collection at 80 K was performed on the smallest single crystal described in Section 6.1.1. Data analysis using *Crysalis^pro^* (Agilent, 2014 ▸) gave the following unconstrained parameters: *a* = 5.2302 (2) Å, *b* = 6.5427 (5) Å, *c* = 11.1823 (4) Å, α = 89.900 (5)°, β = 89.984 (3)°, γ = 89.979 (5)° and wavevector **q** = 0.0000 (8)a* + 0.2502 (11)b* + 0.0000 (13)c*. As discussed in Section 6.1.2, the position of the modulation vector changes with temperature starting from an incommensurate position and it is locked at **q** = (1/4)b* at 130 K. The crystal structure will therefore be described as a commensurately modulated structure. In view of these observations, the modulation is discussed within the framework of a (3+1)-dimensional superspace description, retaining the commensurate character observed in the present dataset. The conditions limiting the reflections at 80 K, *h*0*l*: *l* = 2*n* and *hk*0: *h* + *k* + *m* = 2*n*, are in agreement with a *c* superglide mirror perpendicular to the *b* axis and an 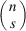 superglide mirror perpendicular to the *c* axis, respectively, and are compatible with the superspace groups *Pmcn*(0σ_2_0)00*s* and *P*2_1_*cn*(0σ_2_0)00*s*. The centrosymmetric superspace group was chosen since the space group of the high-temperature form is *Pmcn*.

The data were integrated using *Crysalis^pro^* (Agilent, 2014 ▸); only main [911 with *I* ≥ 3σ(*I*)] and first-order satellite [1343 with *I* ≥ 3σ(*I*)] independent reflections were observed. Corresponding to the superspace group *Pmcn*(0σ_2_0)00*s*, the average unit cell contains six independent atoms (one W, one P and four O atoms). A periodic modulation function expressed as a Fourier expansion developed up to the first order was introduced to describe the possible atomic displacements of W. The refined atomic displacements are relatively moderate (0.43 Å along **y** and 0.87 Å along **z**) but significant; the *R* factor for the satellite reflections dropped to 17.3%. One displacive modulation wave was then introduced for P and O atoms; the *R* factor for the satellite reflections reached 6.1%. The atomic displacements observed for phosphorus and oxygen are quite large both along **y** and **z** (up to 1.06 Å for P and 2.11 Å for O).

At this step of the refinement, different sections of the superspace were analyzed. Two special sections and a general one can be identified:

(1) section 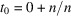 with the space group *P*2_1_/*m*11 (unique axis *a*) for the (**a**, 4**b**, **c**) supercell;

(2) general section 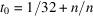 with space group *Pm* (unique axis *a*) for the (**a**, 4**b**, **c**) supercell; and

(3) section 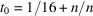 with the space group *Pmc*2_1_ for the (**a**, 4**b**, **c**) supercell.

These three sections were tested, *i.e.* choosing the commensurate option in *Jana2020* (Petříček *et al.*, 2014 ▸) the structure refinement was performed using three possible *t*_0_ origins along the fourth dimension. A new integration using *Crysalis^pro^* (Agilent, 2014 ▸) but with the 2/*m*11 Laue class was performed to check the monoclinic sections. Furthermore, for the monoclinic sections twin domains related by a twofold axis parallel to **c** were introduced and their relative proportion was refined. Consequently, depending on the *t*_0_ section, the refinement results were:

(1) section 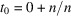, monoclinic symmetry with unique axis *a*, with the space group *P*2_1_/*m*11 (centrosymmetric): 1664 independent main reflections with *I* ≥ 3σ(*I*), *R*0 = 2.65%, 2369 independent first-order satellite reflections with *I* ≥ 3σ(*I*), *R*1 = 6.52%;

(2) section 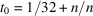, monoclinic symmetry with unique axis *a*, with space group *Pm* (noncentrosymmetric): 2962 independent main reflections with *I* ≥ 3σ(*I*), *R*0 = 2.85%, 3867 independent first-order satellite reflections with *I* ≥ 3σ(*I*), *R*1 = 7.03%; and

(3) section 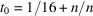, orthorhombic symmetry, space group *Pmc*2_1_ (noncentrosymmetric): 1686 independent main reflections with *I* ≥ 3σ(*I*), *R*0 = 2.62%, 2318 independent first-order satellite reflections with *I* ≥ 3σ(*I*), *R*1 = 6.49%.

The refinements were performed in a parallel way for the three sections. They converged quickly toward stable solutions and no anomalies in either ADPs or chemistry (interatomic distances or angles) were observed. Unfortunately, the differences in the agreement factors for the analyzed sections were too small to conclude in favor of one solution or another.

At this stage, we digress in order to discuss the driving force of the modulation and the intensity of the satellite reflections. The strongest atomic displacements were clearly affecting the O atoms as shown in Fig. S4, but the W atomic displacements are too large to be negligible. Fig. S5 gives an overview of the main atomic displacements via the observation of the Lissajous trace of the modulation for each atom. As confirmed by the values of the different angles and distances (Table S7), the impact of these modulations on the PO_4_ and WO_6_ polyhedra is very limited. The W—O and P—O distances show variations smaller than 0.02 Å and in the same way analysis of the O—W—O and O—P—O angles supports the virtual absence of polyhedra distortions. Thus, the PO_4_ and WO_6_ polyhedra appear to be very rigid.

**Figure 4:**
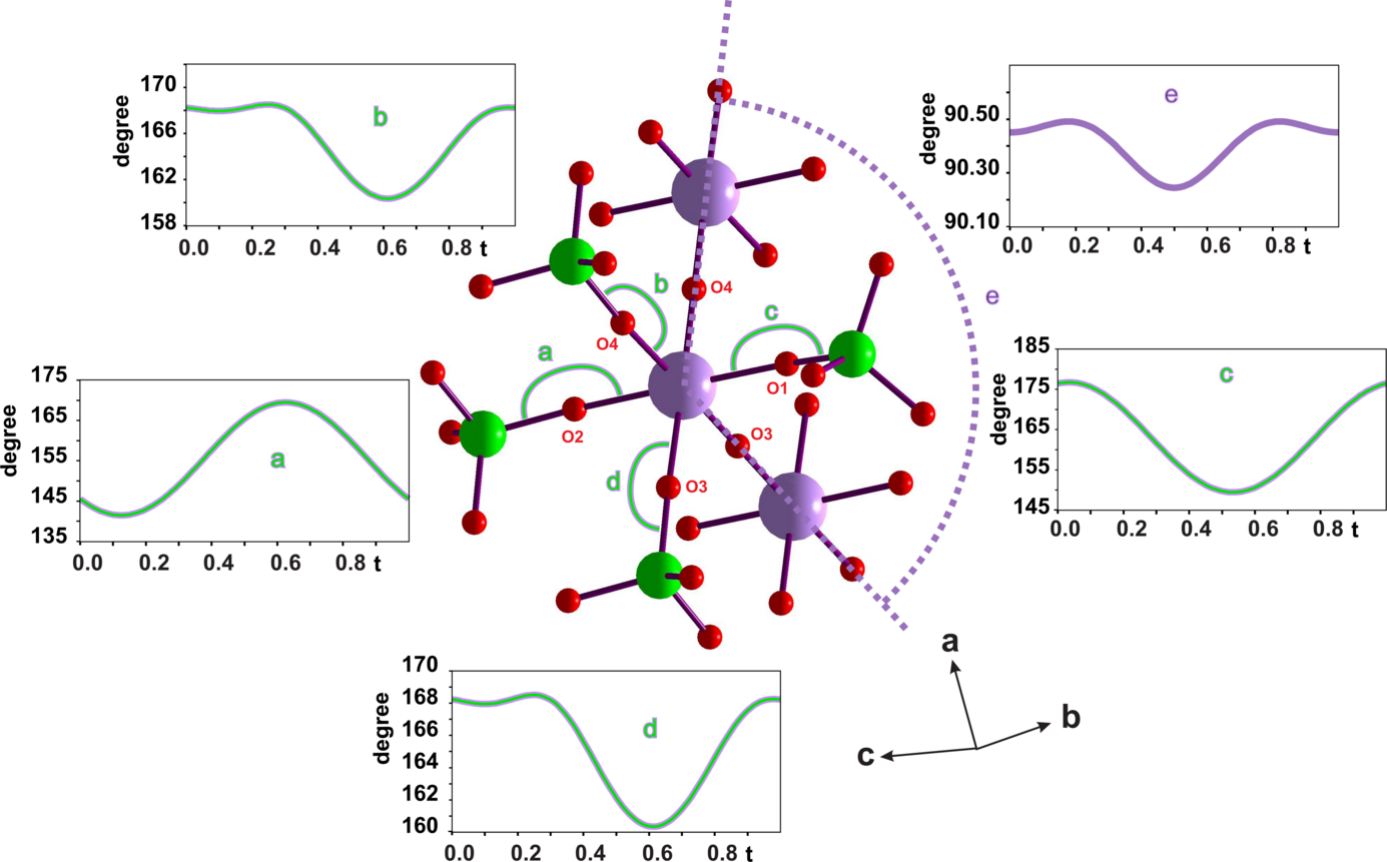
Segment of the (WO_6_)_∞_ chains. The evolution versus *t* of the angles labeled a, b, c, d and e in the figure are plotted. W, P and O atoms are drawn using purple, green and red, respectively.

Investigation of the P—O—W and W—O—W angles was more instructive for visualizing the main effect of the modulation. Fig. 4 ▸ summarizes the deviations of these angles from their average values. As shown by the very small variation of the W—O—W angles, the zigzag chains of edge-sharing WO_6_ octahedra shown in Fig. 1 ▸(*b*) keep the same structure in the fundamental state and below the transition.

On the contrary, the P—O—W angles denoted b and d show a moderate variation of roughly 8° and the a and c angles show a very strong modulation of about 25°. All these results show that the main effect of the modulation is the strong tilting of the PO_4_ polyhedra around the octahedral infinite chain, which remains globally unchanged.

**Figure 5:**
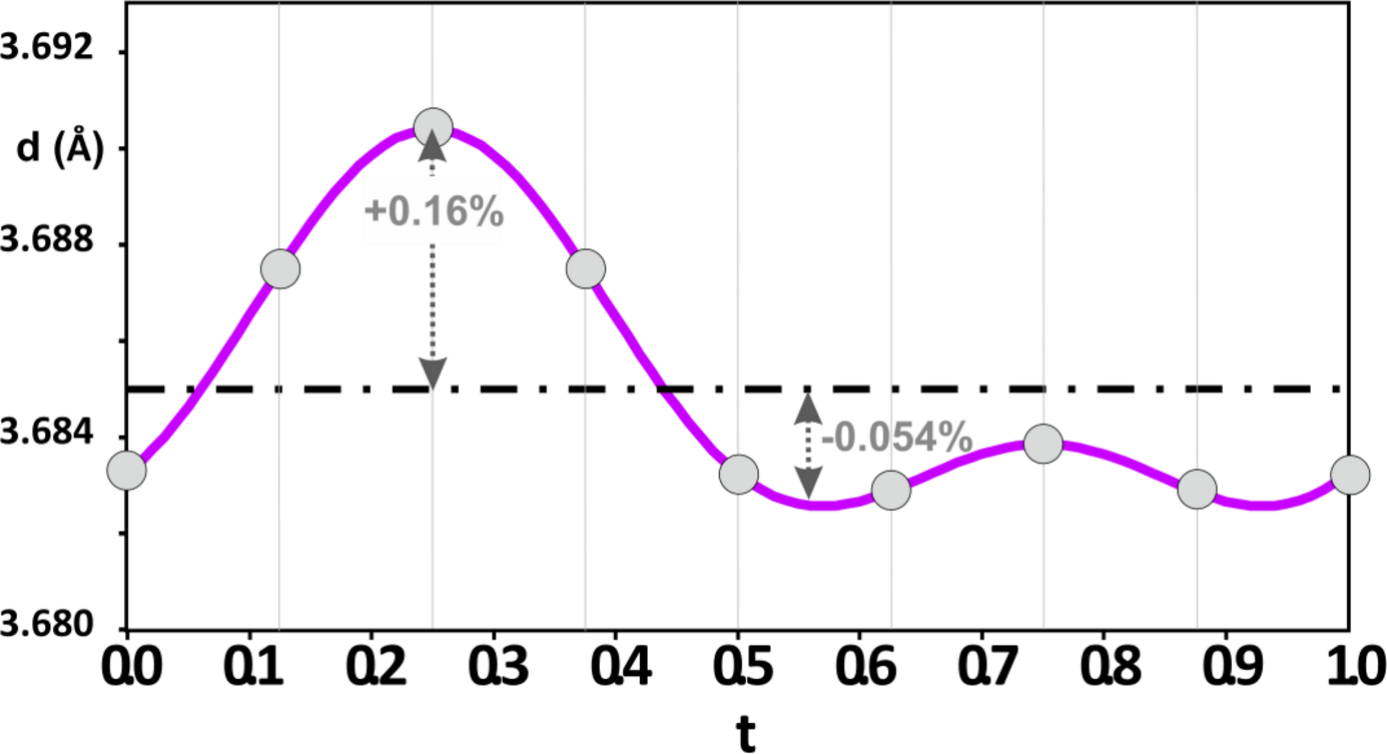
Proposed evolution versus *t* of the W–W first-neighbor distances within the (WO_6_)_∞_ chains. The corresponding distance in the RT structure is drawn using a dotted-dashed line, calculated with the cell parameters at 80 K to limit the effect of thermal expansion. Vertical lines show the relevant distances for the *t*_0_ = 0 monoclinic section.

Let us now go into the detail of the analysis of the (WO_6_)_∞_ chains. Fig. 5 ▸ shows the evolution of W1–W1 distances as a function of *t*; in the case of a commensurate modulated structure only certain points of the presented curve have physical meaning; they derive from the selection of the section of the superspace (where *t*_0_ = 0). Each of the eight points reported in gray in Fig. 5 ▸ corresponds to the W1–W1 distance in one of eight different infinite chains. We can see that at 80 K 62.5% (5/8) of the infinite chains have contractions of W1–W1 distances of the order of 0.05% compared with the distances observed in the fundamental state, and 12.5% (3/8) reveal expansions of between 0.10% and 0.16%. It should be noted that the observed contractions are of the order of magnitude of that reported in the case of the setting up of a charge density wave.

#### 6.1.4. Description of the structural model: analysis in the 80 K ≤ *T* ≤ 285 K interval

**Figure 6:**
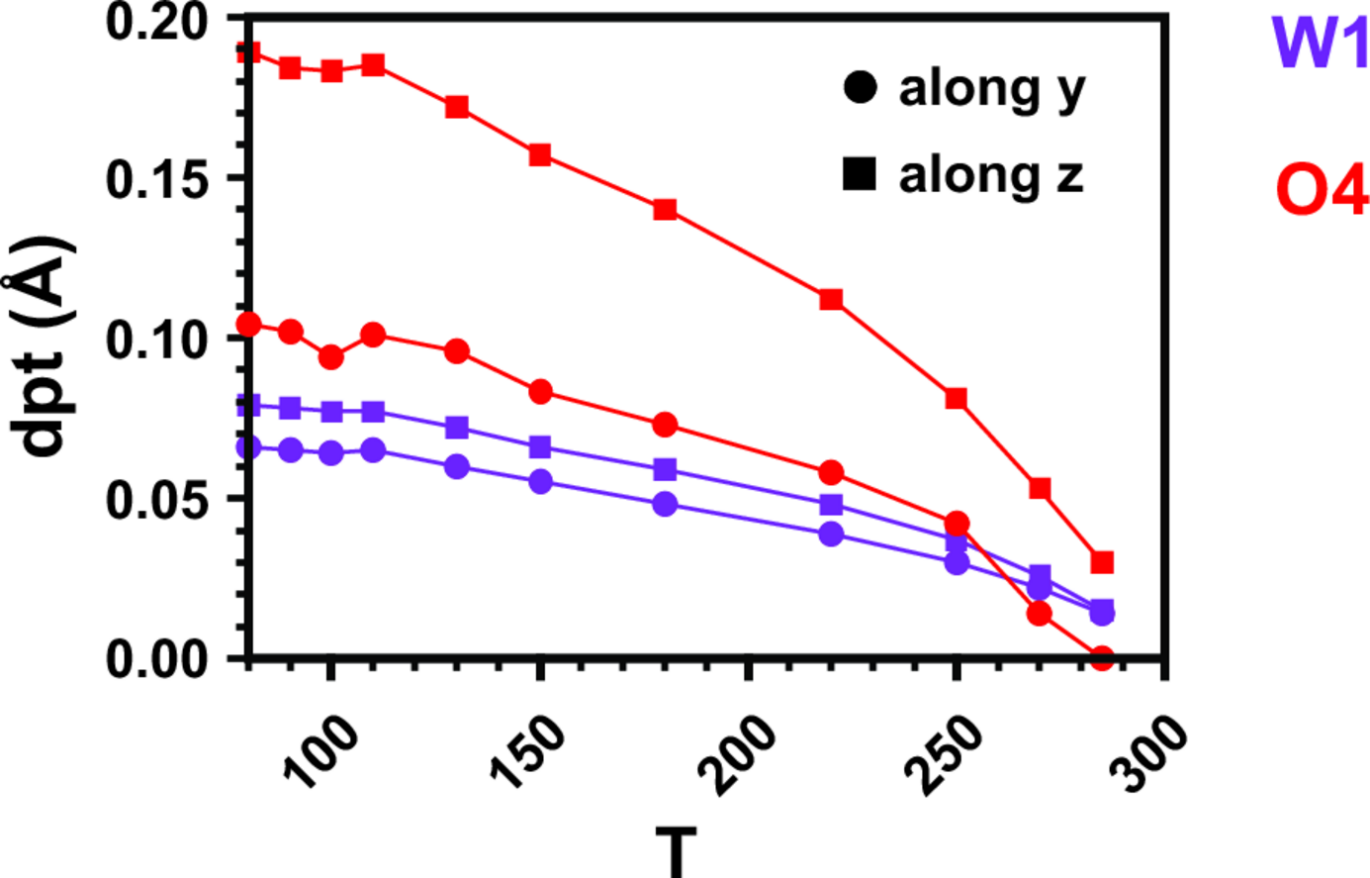
Evolution of the maximum atomic displacements (dpt) extracted from Fig. S4 for W1 (purple) and O4 (red) versus *T* (in K).

Following the study carried out at 80 K, an analysis of a sampling of the data measured between 80 K and 285 K was carried out. The aim of this analysis was to show the impact of decreasing temperature on the modulation and especially the maximum atomic displacements, and then to follow the modulation versus *T*. The data at 80, 110 and 150 K were collected up to sin(θ_max_)/λ = 0.832 Å^−1^; data sets at 130, 180, 220, 250, 270 and 285 K were limited to sin(θ_max_)/λ = 0.624 Å^−1^. The different parameters used to validate the structural refinements were satisfying. Nevertheless, the reliability factor calculated for the first-order satellite reflections at 285 K was relatively high (∼26%). But, as previously reported in Section 6.1.2, the intensity of the satellite reflections decreases when the temperature increases and for this data set only a few satellites with very weak intensity are observed (120 first-order satellites corresponding to 1.4% of the overall diffracted intensity at 285 K against 881 first-order satellites corresponding to 22.8% of the overall diffracted intensity at 130 K). Be that as it may, the main characteristics of the modulation can be followed at the different temperatures. Fig. 6 ▸ shows the evolution of the atomic displacements for the tungsten and one of the oxygen atoms; it can be seen that both along **y** and **z** the displacement increases as *T* decreases. The maximum displacements for these two atoms can be extracted at the different temperatures; they are plotted in Fig. 6 ▸. The shapes of the different curves are very similar to the evolution of the satellite intensity versus *T* (Fig. 2 ▸).

### 6.2. MD simulations and symmetry analysis

The molecular dynamics (MD) simulations show interesting results, especially for the high-temperature phase. We introduce the concept of axis librations, which will be useful for understanding the following part. The libration is the oscillation of the axes of rotation with an inclination angle linked to the ‘normal’ axes. The librations are considered for the fourfold axes of the tungsten octahedra, as they were found to be the most prominent movements. The three rotational axes of the octahedron are called yaw, roll and pitch, and the first two are described in Fig. 1 ▸(*d*). The yaw axis runs parallel to the [100] direction, whereas the pitch and the roll axes are in the *YZ* plane. The pitch axis is parallel to the [110] axis and the roll axis is perpendicular to it. Those oscillations describe a preponderant displacement of the oxygen atoms of the octahedra.

**Figure 7:**
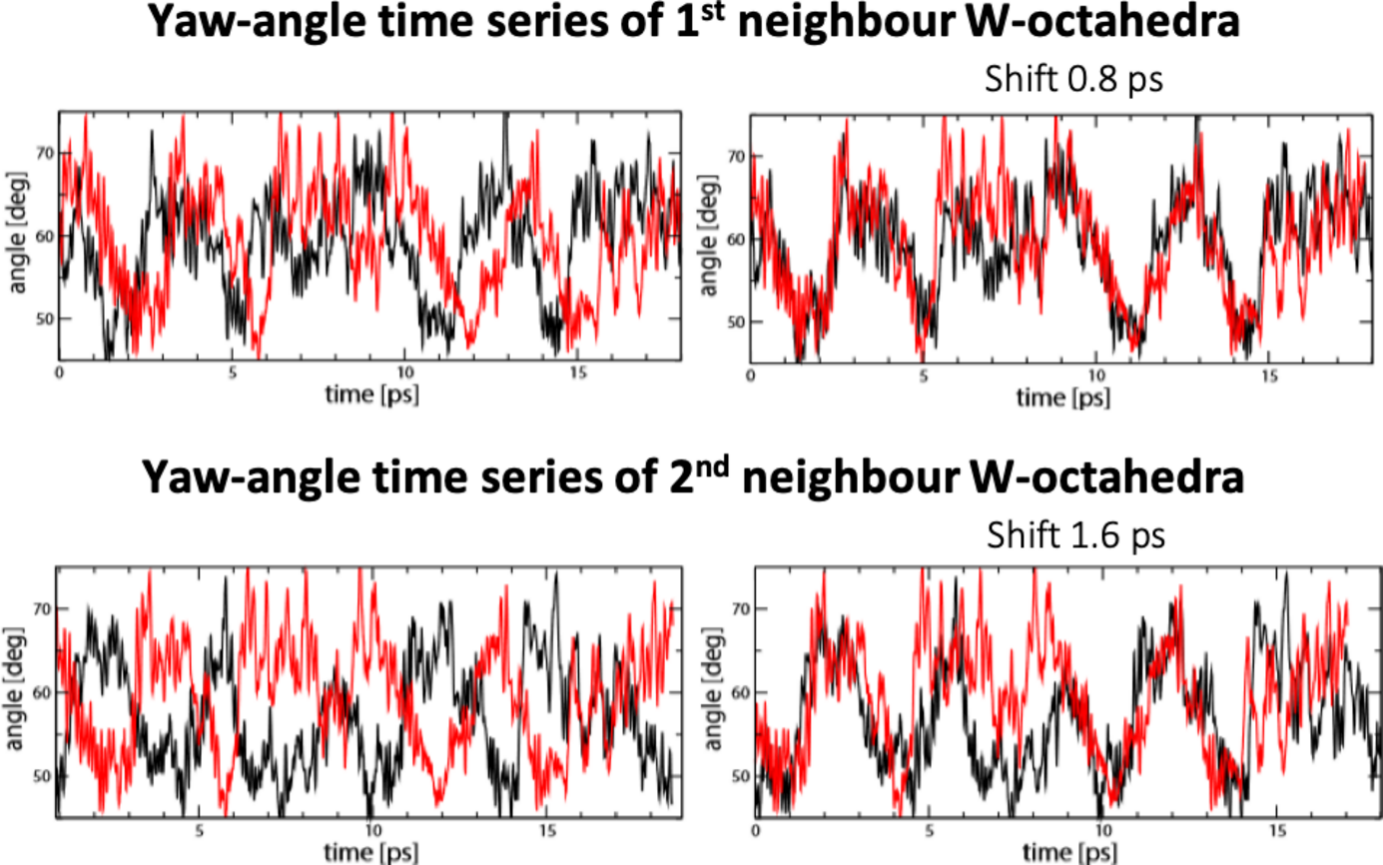
Time dependence of the yaw-angle values for the two octahedra (one shown in red and one in black) of different chains along **b** from the MD simulations at 300 K. Top: the angle versus time series on the left, and after a time delay on the right, of the octahedra between first-neighbor chains. Bottom: the angle versus time series on the left, and after a time delay on the right, of the octahedra between second-neighbor chains. A shift is applied to show the high correlation between these time series at 0.8 ps and 1.6 ps, respectively. The first- and second-neighbor octahedra are marked with the numbers 1, 2 and 3 in Fig. 1(*b*), while the yaw-axes libration and its large angle of libration can be visualized in Fig. 1(*d*).

The superstructure modulation of the CDW phase can be described by the yaw-axis libration, which is the predominant oscillation. The librations last 3–4 ps with a magnitude of ∼12° of the average angle, which is about 62° with respect to the [001] direction. Compared with the other two modes, it is larger and slower.^4^ In fact, the octahedron rotation around the [100] axis has a change of phase by 2π/4 with respect to the neighboring W octahedron in the [010] direction. The rotations in time and space are shown in Fig. 7 ▸ for the simulation at room temperature. The scheme contains the yaw-angle-libration time series of the W octahedra related by (010) and (020) translations. With the octahedra connected by a (010) translation, a high correlation is seen when applying a 0.8 ps shift, whereas for the two connected by a (020) translation, the shift is doubled. This demonstrates that the yaw-axis vibrations break the (010) translation period. This result perfectly explains the fourfold supercell, and we can also conclude that the principal degree of freedom is based on the rigid-body rotations of octahedra, which corroborates the diffraction results.

**Figure 8:**
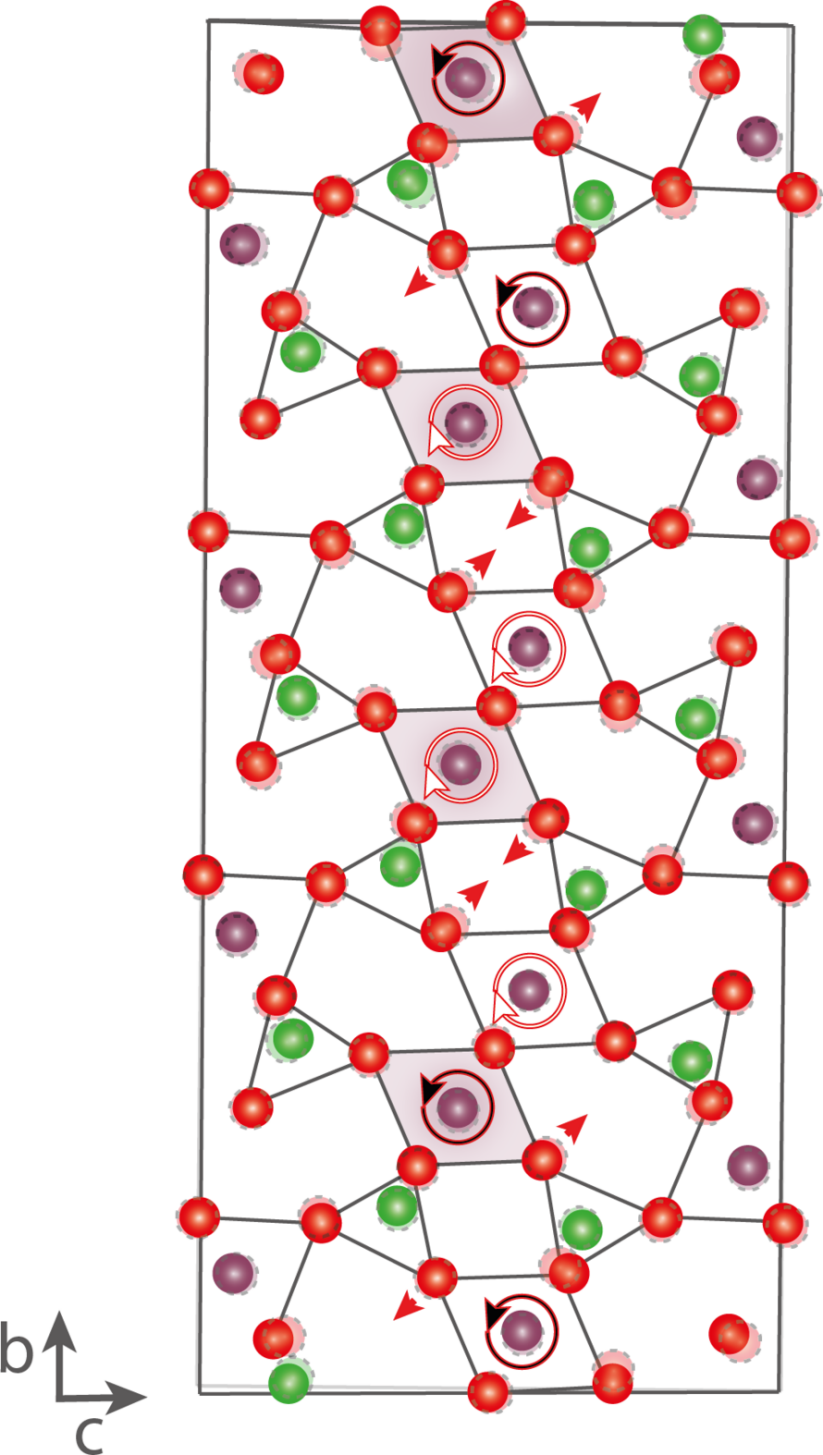
The two principal modes of the ‘order–disorder’ structural phase transition, LD4 and GM1. The LD4 mode can be seen as an anti- and clockwise movement of the octahedra, drawn with black and white circular arrows, respectively. The GM1 mode is an opposite pair movement of the oxygen atoms (straight red arrow). The modes were found by *ISODISTORT* (Campbell *et al.*, 2006 ▸; Stokes *et al.*, 2010 ▸) looking at the structure refinements for the high-temperature phase and the CDW ground state, a monoclinic structure.

With those results to add to the diffraction results shown before, the global picture seems to consider the structural phase transition as ‘order–disorder’. The structure at high temperature is almost orthorhombic and, as reported earlier from the diffraction results, it transforms at the CDW ground state to a monoclinic phase. Two modes describe the transition, LD4 and GM1. The first one is the primary mode with a *k*-vector along b*, and the latter is coupled with the primary mode. The two modes are described in Fig. 8 ▸. Choosing the monoclinic CDW ground-state option, the two modes create the movement as described from the MD simulations, the so-called axis librations (which is exactly the sum of the two modes found by the rigid model). This movement can be seen as a strong atomic displacement of the oxygen and phosphate atoms. It describes the rigid-body movement of the octahedra driven by the freedom of the oxygen atoms. The rigid-body motion gives rise to a little readjustment of the tungsten-atom position.^5^

### 6.3. Dynamics-related measurements (diffuse scattering and inelastic X-ray scattering)

**Figure 9:**
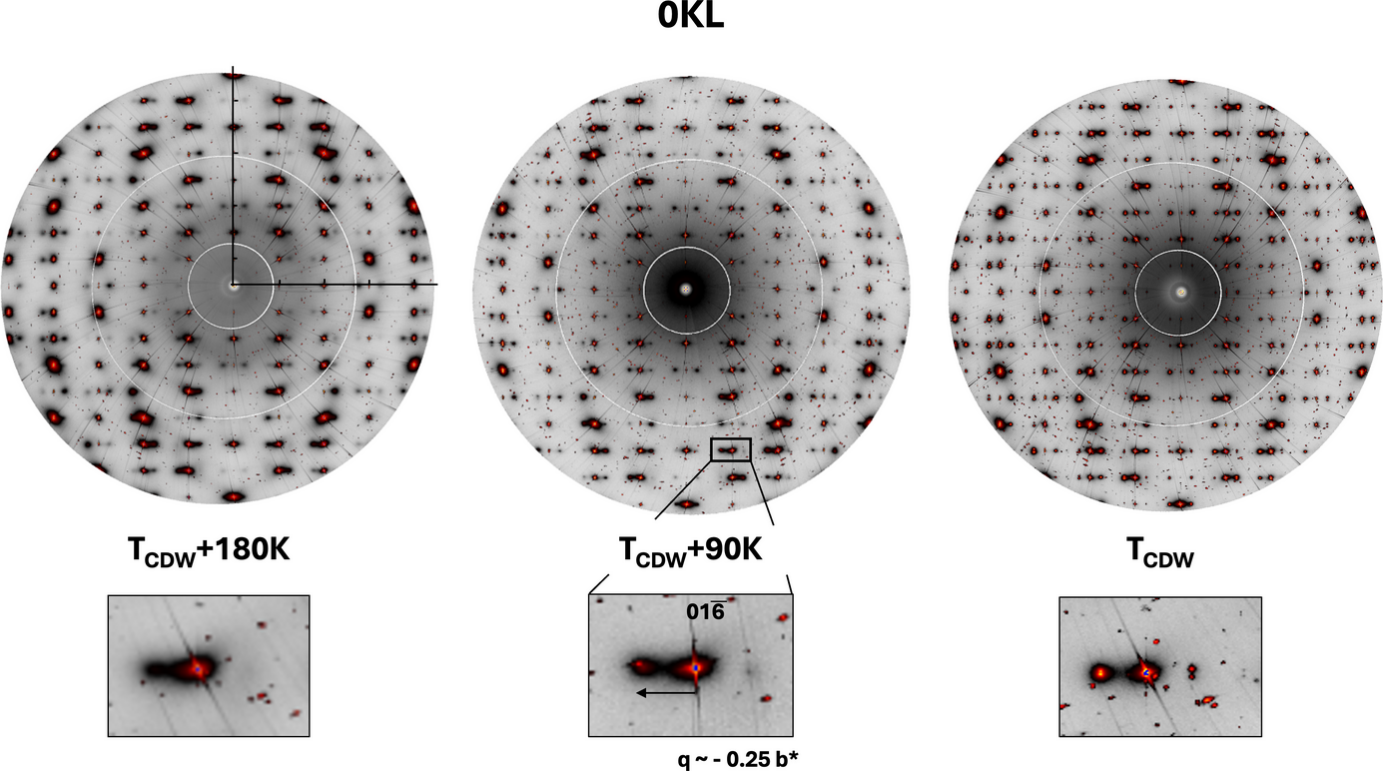
Diffuse scattering 0*KL* maps at different temperatures, above the transition temperature (*T*_CDW_ + 180 K and + 90 K) and at the transition temperature. Insets focused on 016 show the part of reciprocal space studied further by inelastic scattering. They also show the diffuse scattering and Bragg-peak satellite at **q** ≃ −0.25b*.

**Figure 10:**
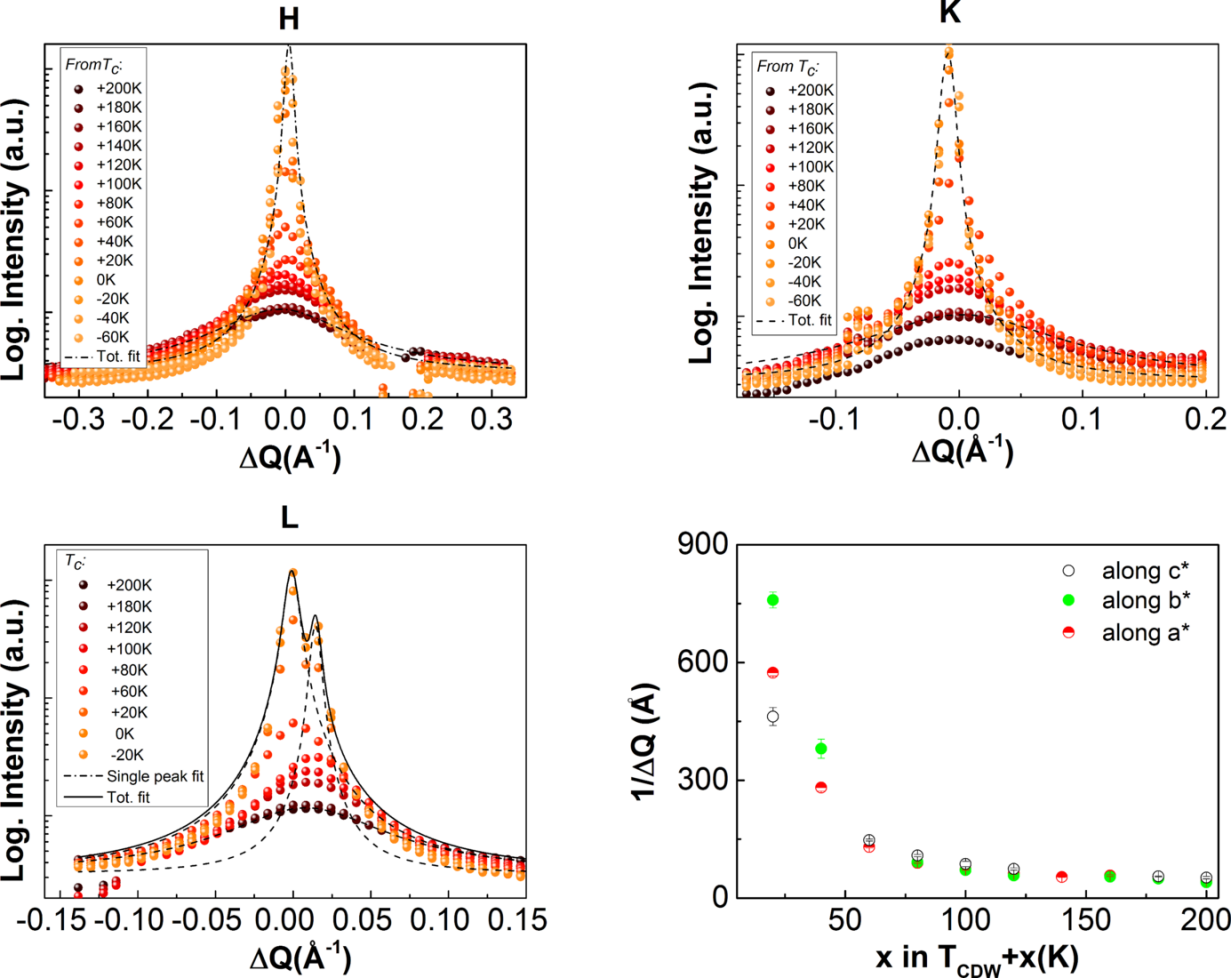
From top left to bottom right: The projections on the three directions around 0 0.75 5 above and below the transition. The experimental data are shown by dots, whereas the fitting of two temperatures are shown by dotted lines, as an example of the high-*T* phase (*T*_c_ + 200 K) and of the CDW ground-state phase (*T*_c_ − 20 K) peaks. In c*, twinning was considered, with the distance and intensity ratio between the two kept constant. The temperature dependence of 1/Δ*Q* on the three directions shows the isotropic shape of the diffuse pattern, which develops complete long-range periodic order at the transition temperature.

Inelastic X-ray scattering measurements were carried out on the high-symmetry phase approaching the CDW ground state, in order to reveal the Kohn anomaly and the phonon behavior linked to the electronic/structural instability. The maps of diffuse scattering on the (*H*0*L*) plane show a temperature dependence, where a pre-transitional diffuse pattern is isotropically distributed around the **Q**-point related to the satellite of the CDW phase, as shown in Fig. 9 ▸. In fact, the distribution is positioned about the center of the CDW vector, with a b* component of ∼0.25, considering the Bragg node. In order to study the temperature dependence of the diffuse scattering, the chosen **Q**-point for the DS measurement should be completely separated from the Bragg node. Moreover, a weak Bragg peak does not present any strong thermal component and the temperature dependence of the pre-transitional diffuse scattering on the CDW satellite can be better observed. For this reason, we chose the CDW satellite at the position ∼0 0.75 5. The shape is isotropic and the evolution in temperature is similar for all of them, as shown in Fig. 10 ▸.^6^

The projections of the diffuse scattering in the three directions permit the evaluation of the temperature dependence of 1/Δ**Q** approaching the transition (Fig. 10 ▸). This can be extracted with a fit of the peak through a Voigt function, where the instrumental resolution is described through a Gaussian profile and the diffuse scattering through a Lorentzian. The background is fixed with a constant, *y*0, which was taken by averaging a segment of reciprocal space where no Bragg or diffuse scattering are present. From the Lorentzian full-width at half-maxima (FWHMs), we confirm similar behavior in the three directions, reaching the resolution function around the transition temperature, as shown in Table 1 ▸. As expected, at the transition temperature, the diffuse scattering signal becomes completely elastic following the periodicity of the full crystal. Thus, the satellite peak is shaped in each direction.

Because of the temperature shift caused by sample heating during the diffuse scattering measurements (approximately 100 K), additional rocking curve scans were performed using the long-arm spectrometer at beamline ID28. The rocking curves collected at various temperatures are presented in Fig. S7. From these measurements, we confirm that the elastic component of the CDW phase clearly emerges around 290 K. The data-fitting procedure follows the same approach used for the diffuse scattering analysis. In the pre-transitional state, the satellite peak is well described by a Lorentzian profile. As the system approaches the transition temperature, the instrumental resolution, modeled by a Gaussian profile, must be taken into account. For this reason, a pseudo-Voigt function is used in this intermediate regime. Below the transition temperature, the peak shape is dominated by the instrumental resolution, and a Gaussian profile is sufficient to describe the data. The FWHMs of the fitting at different temperatures are reported in Fig. S8. The transition temperature derived from this analysis is approximately 280 K, about 10 K lower than the value obtained from diffraction measurements. This discrepancy may arise from instrumental uncertainties, as both measurements were conducted using a cryogenic gas stream.

**Figure 11:**
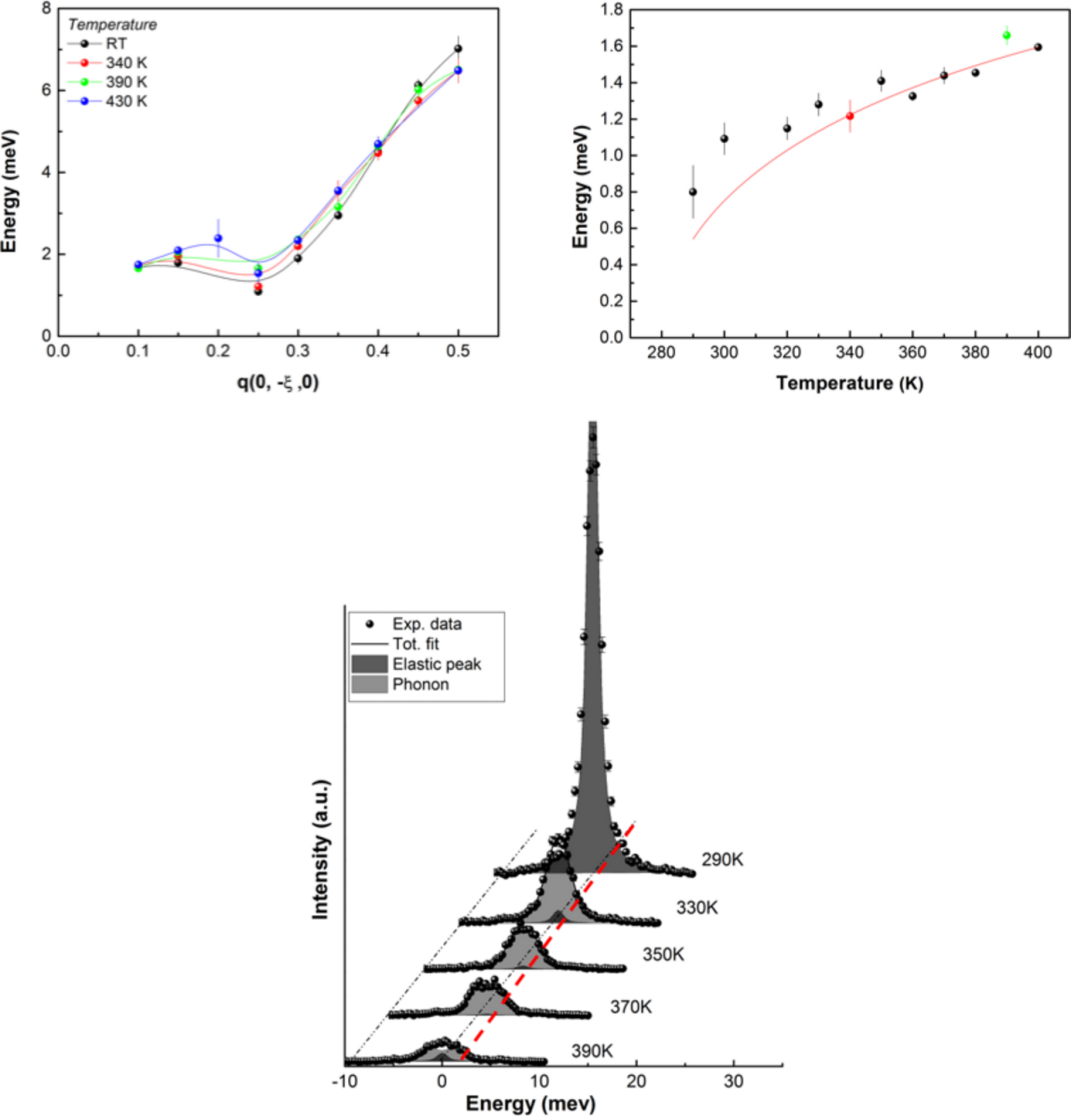
Top left: the full dispersion from the Bragg peak to the border of the Brillouin zone, at the position Y, in the negative direction [0−ξ0]. Top right: the temperature dependence of the energy in the satellite position is shown. Some of the IXS scans on 0 0.75 6 are shown in the figure at the bottom, with the fitting. In the latter, we can see the strong elastic component already at 290 K.

Following the position of the CDW and the pre-transitional diffuse pattern, the phonon dispersion along the b* direction from the 016 Bragg peak was measured through IXS. In this case, to measure the dynamic component, we chose an intense Bragg peak. This ensured that we observed intense phonon branches simultaneously. The ROI (region of interest) in reciprocal space can be seen in the magnified inset of the DS maps at three different temperatures in Fig. 9 ▸. The satellite is condensing, and a clear phonon softening is present at **q**_CDW_. The full dispersion was measured at four different temperatures on cooling the system, as shown in Fig. 11 ▸(*a*). The temperature dependence of the phonon energy is reported in Fig. 11 ▸(*b*), where the single inelastic scans were recorded from 390 K to 290 K, Fig. 11 ▸(*c*). The energy drops from *E* = 1.66 (50) meV at 390 K to *E* = 0.80 (15) meV at 290 K. At lower temperatures, the elastic contribution is too strong, hiding the inelastic signal, as shown in Fig. 11 ▸(*c*). Expecting the transition at ∼280 K and using a power law function, the phonon does not seem to soften to zero energy. The fitting function is *E* = *E*_0_(1 − *T*_CDW_/*T*)^γ^ to the power of 0.5, as suggested for a second-order transition within the mean-field theory (Chaikin & Lubensky, 1995 ▸; Scott, 1974 ▸), and *E*_0_ corresponds to the high-*T* energy limit. The fitting function is described by the red line in figure Fig. 11 ▸(*b*). The behavior does not follow the predicted temperature dependence of a Kohn anomaly with a complete freezing of the vibration as found for other CDW systems (Hoesch *et al.*, 2009 ▸; Pouget *et al.*, 1985 ▸; Pouget *et al.*, 1991 ▸).

### 6.4. Electronic transport properties and relevance of a CDW

**Figure 12:**
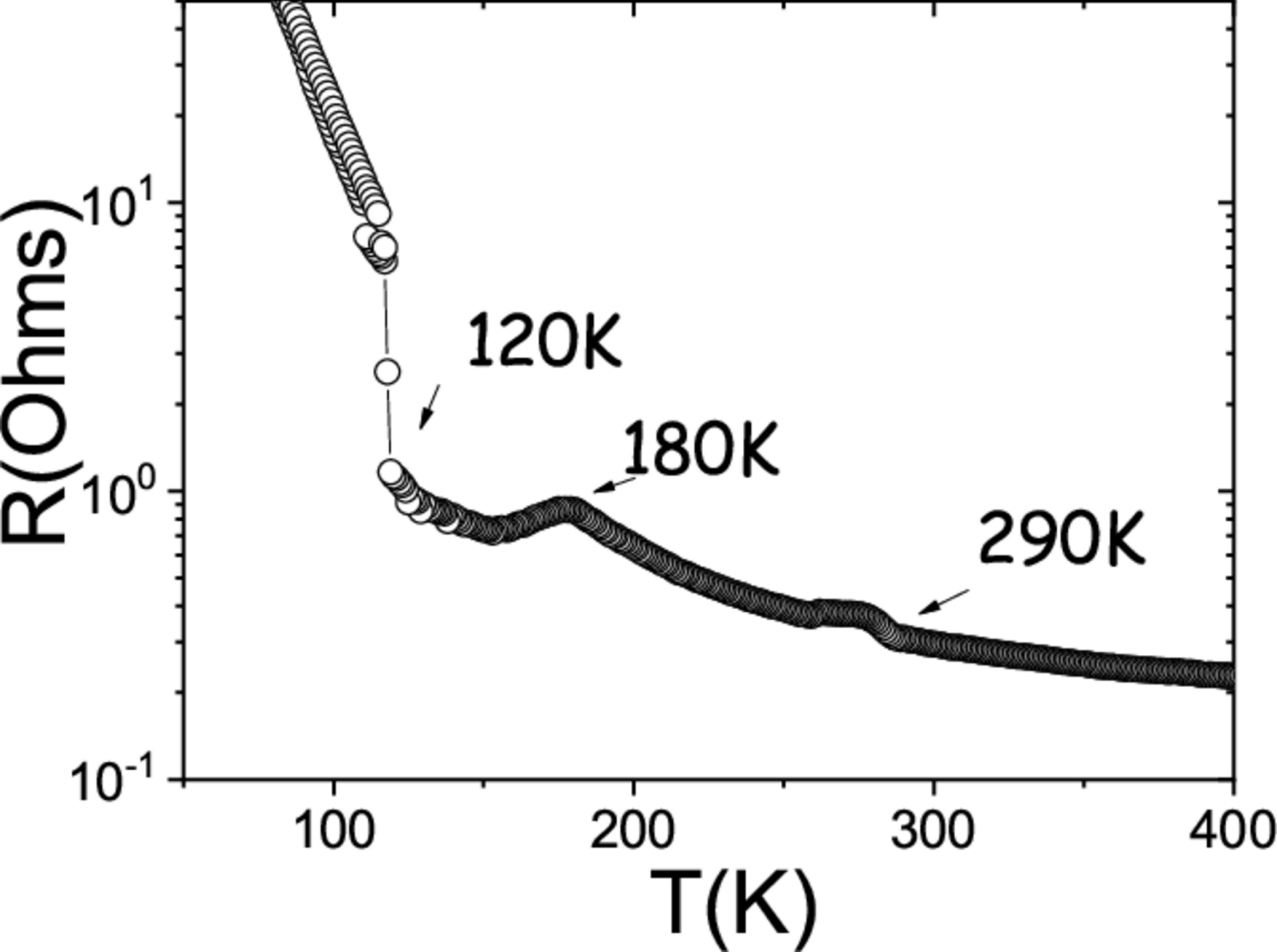
Resistivity versus temperature measured with a biased current with *I* = 1 mA.

Previous measurements have shown a semiconducting-like resistivity in the 50–400 K range (Teweldemedhin *et al.*, 1991 ▸), with no evidence of electronic instability. Our new data indicate more complex resistive behavior, as reported in Fig. 12 ▸.

**Figure 13:**
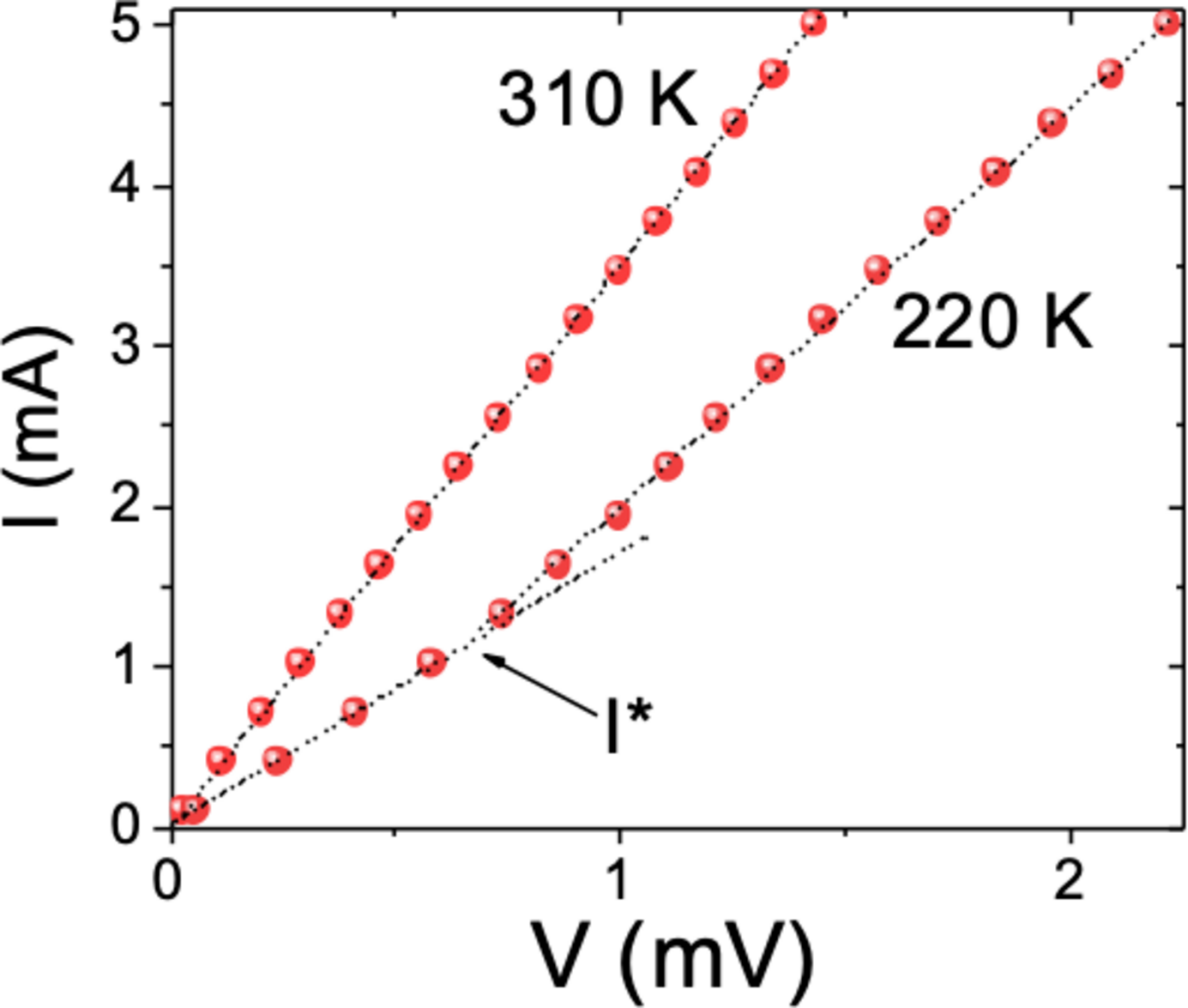
*I*(*V*) curve measured at two different temperatures. Note the ohmic (respectively non-ohmic) behavior at *T* = 310 (respectively 220) K.

When cooling the sample from room to low temperature, measuring with a current of 1 mA, three anomalies in resistivity are observed at *T* ≃ 290, 180 and 120 K. A current-dependence study reveals that the two anomalies at the lower temperatures are strongly shifted by an only moderate current (typically 100–1000 µA). The resistivity shows a dominant non-linear behavior. Since the reported phase transition starts at 290 K, we can see the first anomaly in the resistivity, while the lock-in is shown by the stronger and sharper step at 120 K. We then performed isothermal *V* (voltage)/*I* (current) measurements from 320 K to 70 K with steps of 5 K, using an external setup (current source ADRET 103A, Keithley Nanovoltmeter 2182A). Some typical curves are shown in Fig. 13 ▸.

**Figure 14:**
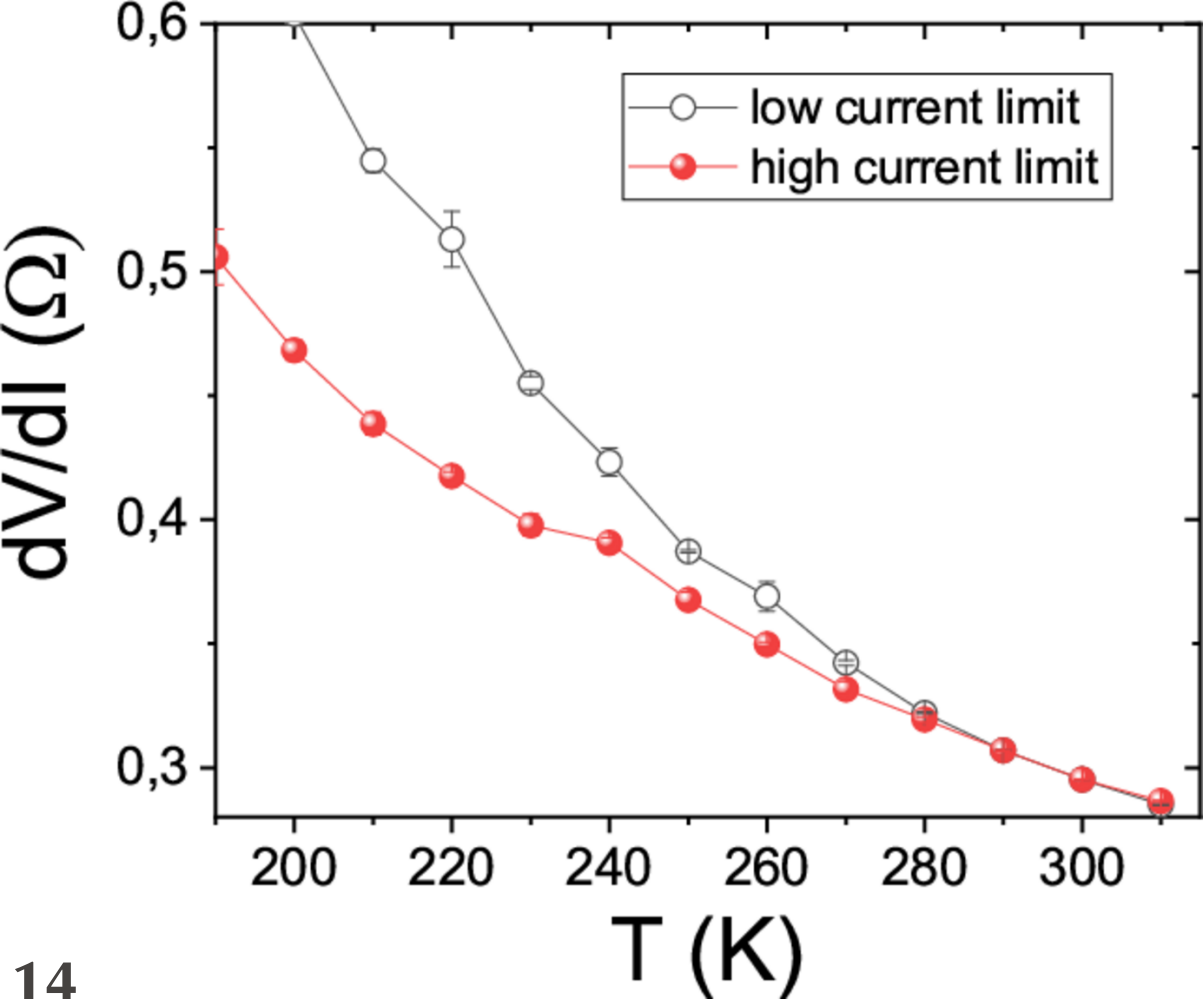
Differential resistance, deduced from the slopes of *V*(*I*) curves at low and high current, from 0 to 1 mA and from 4 to 5 mA, respectively, as a function of temperature. Slopes are different, *i.e.* non-ohmicity appears for *T* between 280 and 290 K.

**Figure 15:**
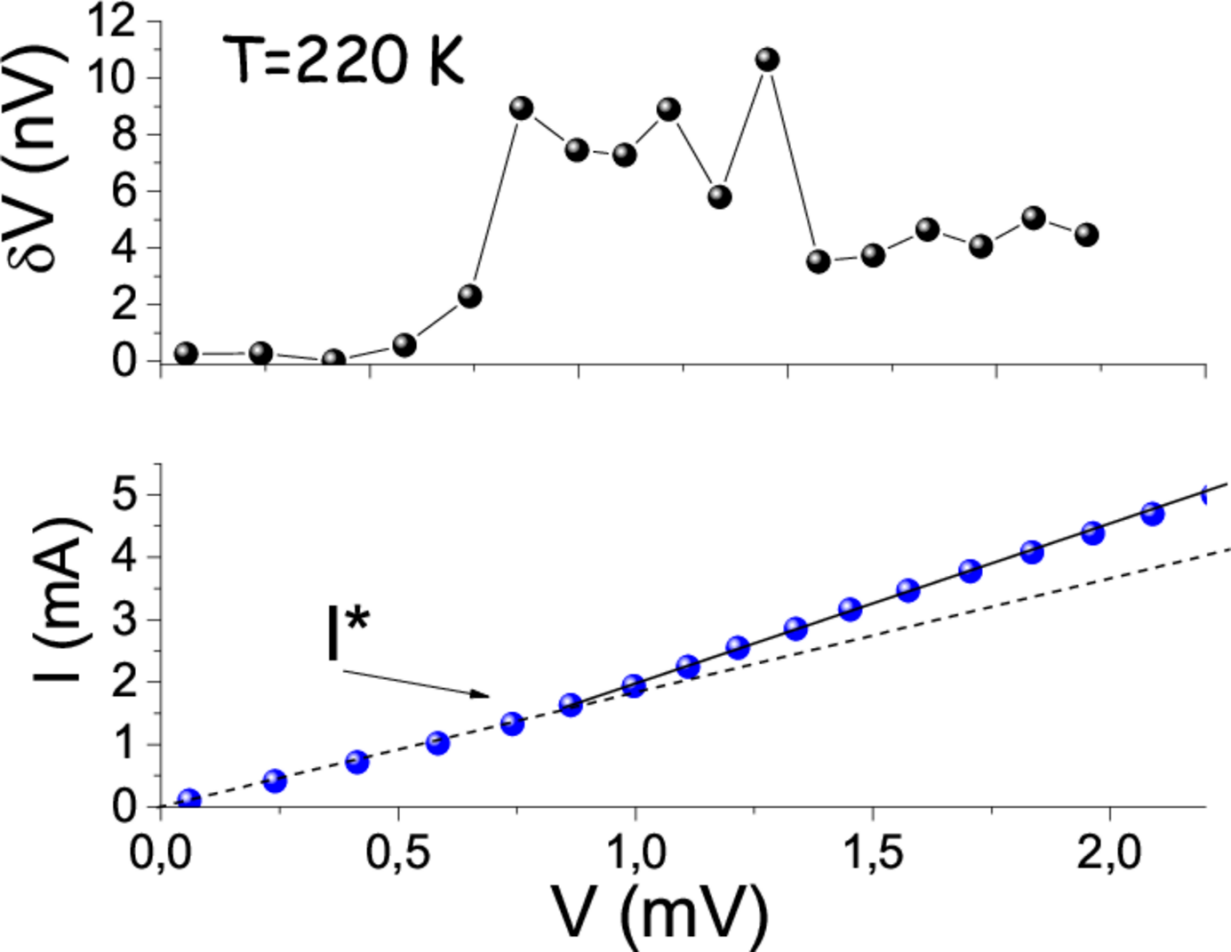
Top: δ*V* as function of applied current, showing a strong increase at *I* ≃ I*, which is typical of a depinning transition. Bottom: *V*(*I*) curve measured at *T* = 220 K, with I* the threshold current marking the non-linear behavior.

In the high-temperature range, the *V*(*I*) curve is that of an ohmic, conventional conductor with a single slope *R* = *V*/*I*. At *T* ≲ 280 K, a slight departure from perfect ohmicity can be noted, which becomes clearer as the temperature decreases. At *T* ≲ 240 K, a two-slope regime is obvious and shows an excess of flowing current above a threshold value denoted I*. We fitted the *V*(*I*) slope in the high- and low-current limits to find the temperature where the non-ohmic behavior appears. The values at each temperature are reported in Fig. 14 ▸. Within the resolution, the two slopes differ at temperatures below *T* = 280–290 K. This behavior is characteristic of pinning/sliding collective modes of charge density waves (Monceau, 2012 ▸), as reported for example in the quasi-1D conductor NbSe_3_. In addition to non-linear conductivity, sliding CDWs are expected to generate both low-frequency broadband noise (BBN) (Fleming & Grimes, 1979 ▸), mainly due to the CDW configuration and velocity fluctuations, and high-frequency narrow-band noise (NBN) (Bhattacharya *et al.*, 1989 ▸; Bloom *et al.*, 1993 ▸) due to their harmonic nature. Here, we have not attempted to measure the NBN, but focused on the BBN, which is expected to be, as a general rule for a depinning transition, large close to the threshold field. At a fixed temperature, for each value of (noise-free) applied current, we recorded the autopower spectra *S*_*VV*_(*f*) (V^2^ Hz^−1^) of the voltage noise, then calculated the noise power defined here by δ*V*^2^ = 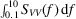. As expected, δ*V*^2^ does not depend on the applied current in the ohmic regime at 310 K and is limited by the experimental resolution of the preamplifier, ∼1 nV (not shown here). On cooling the system, BBN can be observed along with the non-linear conductivity. A typical example is reported in Fig. 15 ▸, where the NBN value is limited by the resolution below I*, then increases sharply at I* and tends to be quiet at large currents, in agreement with successive pinning, depinning and sliding regimes. We conclude that the electronic transport properties of (PO_2_)_4_(WO_3_)_4_ change at *T* ≲ 290 K, in the structurally modulated state, and both non-linear DC conductivity and BBN appear. These peculiar electronic properties associated with the periodic lattice distortion are strong experimental evidence for a CDW phase (Monceau, 2012 ▸). It is worth noting that this is the first report of such a sliding collective mode in the MPTB family.

As discussed above, (PO_2_)_4_(WO_3_)_4_ is the only member of the MTBP family where the W atoms are located in zigzag chains, compared with planes for all other members with *m* > 2. Thus, it can be described from its band structure (Canadell & Whangbo, 1990 ▸) as a quasi-1D conductor. We measured here the electric field for depinning in the range of 0.05 V cm^−1^, which is very typical of a (weakly pinned) 1D CDW and with an incommensurate modulation vector. To compare, no evidence of depinning/sliding was found in the quasi-2D bronze K_1.15_P_4_W_8_O_32_ (the *m* = 4 form of MPTBp) with an electric field of 0.12 V cm^−1^ (Kolincio *et al.*, 2016*a* ▸). Our results are then consistent with the idea that a small CDW dimensionality is a key parameter for achieving small depinning thresholds. Another difference in physical properties compared with higher-dimension bronzes is the absence here of a significant magnetoresistance in the CDW state, compared with the enhanced magnetoresistance observed in several others [*i.e.m* = 6, doped *m* = 4 and *m* = 10 (Kolincio *et al.*, 2016*a* ▸; Kolincio *et al.*, 2020 ▸; Kolincio *et al.*, 2016*b* ▸)].

In general, quasi-1D CDWs can be described well by weak electron–phonon coupling scenarios. To better understand this observation, we analyzed the temperature variation of the scattering intensity of the first-order satellite peak as measured by XRD. The idea behind this is that, as far as weak displacements are assumed, this intensity is proportional to the square of the CDW gap, providing a direct link between the electronic and structural degrees of freedom (Grüner, 1988 ▸). In principle, the BCS gap equation and its deduced temperature variation should be solved self-consistently and numerically. However, a very good and practical analytical approximation can be used (Gross *et al.*, 1986 ▸). As shown in Fig. 2 ▸, there is a very good agreement between the experimental data and the gap interpolation formula used in the weak coupling limit.

## 7. Discussion

The member *m* = 2 of the monophosphate tungsten bronze family differs structurally from the rest of the series owing to its isolated zigzag chains of tungsten atoms, rather than the more typical layered W slabs. This distinctive feature makes it the only compound in the family to exhibit a quasi-one-dimensional CDW. The difference is evident from the transition temperature, which is significantly higher (*T*_CDW_ = 290 K) and deviates from the trend observed across the series (Roussel *et al.*, 2000 ▸; Roussel *et al.*, 2001 ▸). The structural simplicity of this system enabled a comprehensive investigation of the charge density wave state.

We have examined the system from multiple perspectives: structurally using diffraction and MD simulations; dynamically through DS and IXS; and electronically *via* resistivity measurements. The results converge to form a coherent picture of the phase transition and its nature.

The first set of observations concern the pre-transitional behavior as the system evolves from the high-temperature phase to the CDW phase. DS around the low-temperature satellite peaks reveals a dynamical origin, confirmed by temperature-dependent IXS measurements. The phonon correlation length diverges near *T*_CDW_, displaying isotropic behavior in reciprocal space. MD simulations show highly correlated displacements of oxygen atoms surrounding the tungsten-atom centers. These displacements are responsible for the low-energy phonon mode, which softens to *E* = 0.80 (15) meV just above the transition temperature (*T*_CDW_ + 10 K) at the corresponding wavevector **q**_CDW_. The softening follows the expected temperature evolution of a CDW system; however, the presence of a strong elastic signal above the transition suggests that the phonon may not fully freeze, masking the inelastic component. In a weak electron–phonon coupling regime, full phonon softening is typically observed (Pouget, 2016 ▸). In contrast, in strong coupling scenarios, no apparent softening is present, and a pre-transitional elastic signal appears before *T*_CDW_, as seen in other systems (Pouget & Canadell, 2024 ▸; Ilakovac *et al.*, 2021 ▸; Subires *et al.*, 2023 ▸). Our results indicate that this compound may exhibit features of both regimes, highlighting the complexity of the transition.

The transition into the CDW phase is also evident in diffraction measurements. The structural distortion primarily involves oxygen atoms, consistent with the phonon and MD analysis. The crystal structure changes from an approximately orthorhombic symmetry (*Pmcn*) to monoclinic (*P*2_1_/*m*). The main symmetry modes involved in this order–disorder transition are LD4 and GM1, as identified through critical wavevector analysis and MD simulations. The latter show that the yaw-angle libration of the tungsten octahedra, related primarily to oxygen displacements, follows a modulation described by a phase shift of 2π/4 between neighboring chains along the *b* direction. Order–disorder transitions are typically associated with strong electron–phonon coupling. However, we note that the monoclinic distortion in the high-symmetry phase is not fully understood, which may affect our interpretation of the transition type. Another interesting feature that distinguishes this member from the rest of the family is that **q**_CDW_ is incommensurate at 290 K and locks in to a commensurate vector upon cooling to 130 K.

Focusing now on the electronic aspects of the CDW, the tungsten atoms exhibit only a weak dimerization along the chain. This is indicative of a weak-coupling CDW, with a gap consistent with BCS theory. The temperature dependence of the satellite peak intensity supports this view. Resistivity measurements reveal a weak anomaly at the primary transition near *T*_CDW_ ≃ 290 K, followed by a much more pronounced step-like feature around 130 K, coinciding with the lock-in of the modulation vector to the commensurate value **q** = 0.25b*. This indicates that while the incommensurate CDW develops at 290 K, the electronic response is significantly enhanced when the modulation becomes commensurate. This behavior is consistent with a progressive stabilization of the CDW state upon cooling. The satellite wavevector **q**_CDW_ therefore evolves from an incommensurate value immediately below *T*_CDW_ to a commensurate position at lower temperature, reflecting a lock-in transition. Interestingly, resistivity data reveal non-ohmic behavior emerging below the transition temperature. This is typically attributed to CDW pinning and sliding modes, and is commonly observed in quasi-1D and 1D conductors.

## 8. Conclusion

At first glance, this system appears to follow a classical CDW instability model, with apparent nesting. The *m* = 2 member of the monophosphate tungsten bronze family undergoes a CDW transition at 290 K, characterized by an incommensurate wavevector of 0.245b* + 0.02c*. This transition is followed by a lock-in at 130 K, characterized by a commensurate modulation wavevector **q** = 0.25b*. DS around the satellite positions reveals an isotropic distribution of low-energy phonons, which condense into Bragg peaks in the CDW ground state.

The structural transition involves tilting of both W octahedra and P tetrahedra, with oxygen displacements reaching up to 0.2 Å. These rearrangements generate strong satellite intensities, but do not significantly affect the tungsten positions along the chain. This suggests a CDW of weak electron–phonon coupling character, further supported by resistivity measurements. The satellite wavevector describes correlations between adjacent W chains, a finding corroborated by MD simulations, which reveal a fourfold periodicity along the *b* axis.^7^

The combination of experimental techniques used in this study provides a comprehensive view of the CDW transition, illustrating its inherent complexity. The new phase is best described by both structural and electronic behavior – yet, in this case, the structural reorganization appears only loosely connected to the electronic CDW. The data suggest that the lattice degrees of freedom play a central role in driving the transition.<!?tpb=-12pt>

## 9. Related literature

The following reference is cited in the supporting information: Hübschle & Dittrich (2011) ▸.

## Supplementary Material

Crystal structure: contains datablock(s) global, CDW, RT. DOI: 10.1107/S2052252526002836/gq5019sup1.cif

Isoviz file for the modes. DOI: 10.1107/S2052252526002836/gq5019sup2.txt

Video of the modes. DOI: 10.1107/S2052252526002836/gq5019sup3.mov

Supporting information. DOI: 10.1107/S2052252526002836/gq5019sup4.pdf

CCDC references: 2547107, 2547108

## Figures and Tables

**Table 1:** Temperature dependence of 1/Δ**Q** along a*, b* and c*

*T* (K)	1/Δ*Q*_a*_ (Å)	1/Δ*Q*_c*_ (Å)	1/Δ*Q*_b*_ (Å)
*T*_c_ + 200	43.0 (6)	40 (2)	52 (3)
*T*_c_ + 120	57 (2)	54 (2)	74 (3)
*T*_c_ + 80	89.6 (1.4)	92 (2)	108 (3)
*T*_c_ + 30	574 (8)	759 (20)	462 (23)

## Footnotes

1 This assumption is supported by the low value of the internal reliability factor observed for the *mmm* Laue class (*R*_int_ = 0.032).

2 The sample used for diffuse scattering and inelastic X-ray scattering is shown in Fig. S1 in the supporting information.

3 Low-intensity reflections that cannot be indexed with the chosen unit cell are observed in the single-crystal X-ray diffraction data. These reflections are attributed to small crystallites attached to the surface of the main crystal, exhibiting a slight misorientation of 2–3° around the *a* axis. Representing only about 3% of the collected reflections, these contributions may influence the cell refinement and could be responsible for the minor deviation of the α angle from the ideal value of 90°.

4 In fact, the roll-axis libration is a few times faster with a short-range correlation and its angle is smaller. The libration of the pitch axis can be seen as thermal noise, since it is high frequency.

5 The modes and the collective movement given by the phase transition can be seen with the *Isoviz* file created by *ISODISTORT*, which can be found in the supporting information.

6 The complete temperature evolution of the satellite in the three directions can be followed in the 0*KL* and the *H*0.75*L* DS maps shown in Fig. S6.

7 Additional details can be found in the doctoral theses of Arianna Minelli and Elen Duverger-Nedellec (Minelli, 2018 ▸; Duverger-Nedellec, 2017 ▸).
