# Supplementary material for: Atomic structure and formation mechanism of a newly discovered charge density wave in the m = 2 monophosphate tungsten bronze
Source: IUCrJ. 2026 May 29;13(Pt 4):441–55. doi: 10.1107/S2052252526002836 (PMC13324605; doi:10.1107/S2052252526002836)
Supplement: Supplementary file 4 [file m-13-00441-sup4.pdf]

IUCrJ

Volume 13 (2026)

Supporting information for article:

Atomic structure and formation mechanism of a newly discovered charge density wave in the *m* = 2 monophosphate tungsten bronze

Arianna Minelli, Elen Duverger-Nedellec, Olivier Perez, Alain Pautrat, Adrien Girard, Johnathan Bulled, Marek Mihalkovič, Marc de Boissieu and Alexei Bosak

Supplementary Information of Atomic structure and formation mechanism of a newly discovered charge density wave in the m=2 monophosphate tungsten bronze

Arianna Minelli,[a,b,c]* Elen Duverger-Nedellec,[d,e] Olivier Perez,[e] Alain Pautrat,[e] Adrien Girard,[f] Johnathan Bulled,[a] Marek Mihalkovič,[g] Marc de Boissieu[h] and Alexei Bosak[a]

[a]*European Synchrotron Radiation Facility, BP 220, F-38043 Grenoble Cedex, France,* [b]*Department of Chemistry, University of Oxford, South Parks Road, Oxford OX1 3QR, U.K.,* [c]*Neutron Scattering Division, Oak Ridge National Laboratory, Oak Ridge, TN 37831, USA,* [d]*Univ. Bordeaux, CNRS, Bordeaux INP, ICMCB, UMR 5026, F-33600 Pessac, France,* [e]*CRISMAT-ENSICAEN, University of Caen Basse-Normandie, CNRS/UMR 6508, 6 Bd Maréchal Juin, 14050 CAEN Cedex 4, France,* [f]*Sorbonne Université, CNRS, MONARIS, F-75252 Paris, France,* [g]*Institute of Physics, Slovak Academy of Sciences, Dúbravskà cesta 9, Bratislava 84511, Slovak Republic, and* [h]*University of Grenoble Alpes, CNRS, SIMAP, 38000 Grenoble, France. E-mail: minellia@ornl.gov*

Appendix 1
Alternative structure model for the fundamental state

The monoclinic model was also considered for the crystal in the fundamental state. The dataset was re-integrated in Crysalis^{pro} assuming monoclinic symmetry, and the unit-cell parameters were refined as follows: $a = 6.5544(4)\mathring{A}, b = 5.22786(18)\mathring{A}$, $c = 11.1913(7)\mathring{A}$, $\alpha = 90.002(4)°$, $\beta = 90.09(5)°$, and $\gamma = 90.000(4)°$. The monoclinic angle β deviates only marginally from 90°. Data reduction resulted in an R$_{int}$ value of 5.8%, with a completeness of 99.9% to a resolution of 0.62 $\mathring{A}^{-1}$, a redundancy of 17, and $\left\langle \frac{F^2}{\sigma(F^2)} \right\rangle = 49.4$. In space group $P2_1/m$, the structure can be described with two W, two P, and eight O atoms in the asymmetric unit.

Refinement converged to R$_{obs}$ = 2.46% for 1725 independent reflections with $I \geq 3\sigma(I)$, with 81 parameters refined. A twin law corresponding to a mirror plane perpendicular to c was applied, and the refined twin fractions are 0.53 and 0.47. However, the anisotropic displacement parameters of four oxygen atoms are not positive definite.

Taken together, these results do not support a monoclinic description of the structure.

Appendix 2
Additional figures

Here are reported few pictures. In Fig. S1, the sample used for DS and IXS measurement at ID28 (ESRF). Fig. S2 shows the high-Q region measured in ID28 that permits to identify the monoclinic distortion. The temperatures are referred to the cryostream, not referred to the temperature of the sample. It is probable that the beam

was heating the sample ∼100K, thus the temperature of transition will be ∼190K in this configuration. Fig. S3 is showing the role of the **q** component along **c*** to explain the experimental diffraction pattern shown in Fig. 3. Fig. S4 shows the evolution of atomic displacements in time for the atoms W1 and O4 at different temperatures. In Fig. S5, the Lissajous traces of the atomic displacements due to the modulation in the low-temperature phase of m=2 as obtained using in-house diffraction are shown.

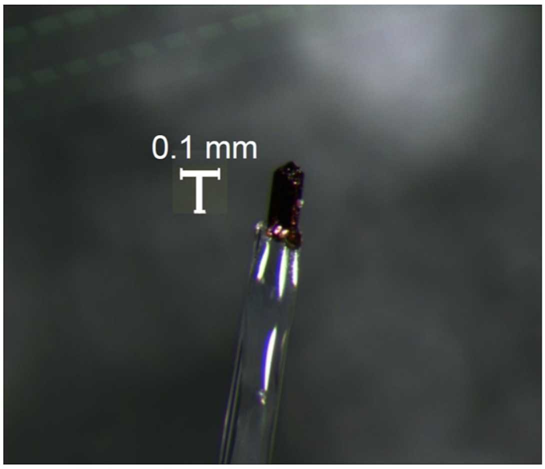

Fig. S1. Picture on the microscope of the m=2 sample with a truncated bar shape, glued on a glass capillary.

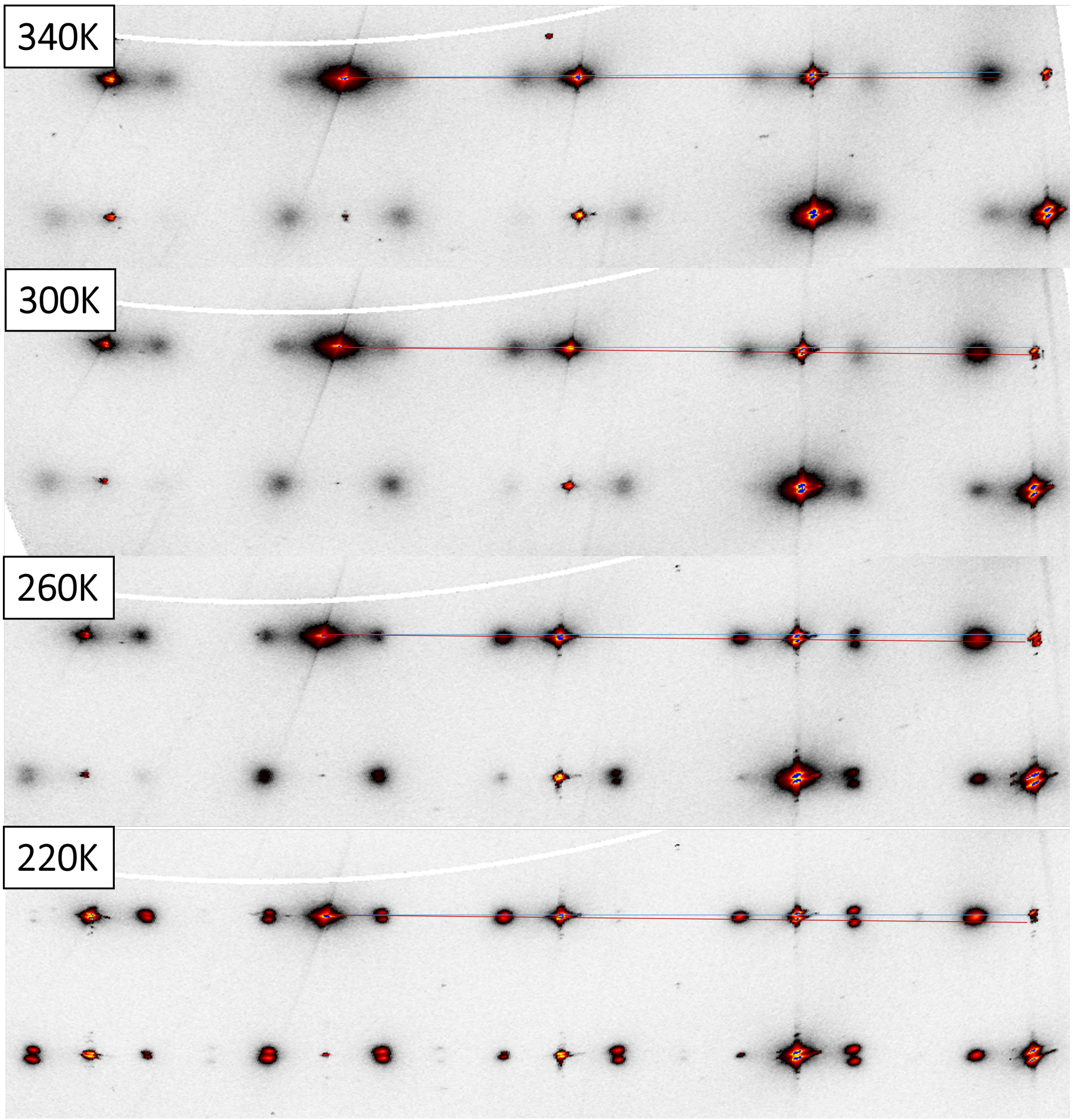

Fig. S2. 0KL diffuse scattering maps above the CDW transition temperature. The red and blue lines follow the two domains showing the monoclinic distortion. The temperatures are referred to the cryostream temperatures, while the sample is probably warmer by about 100K.

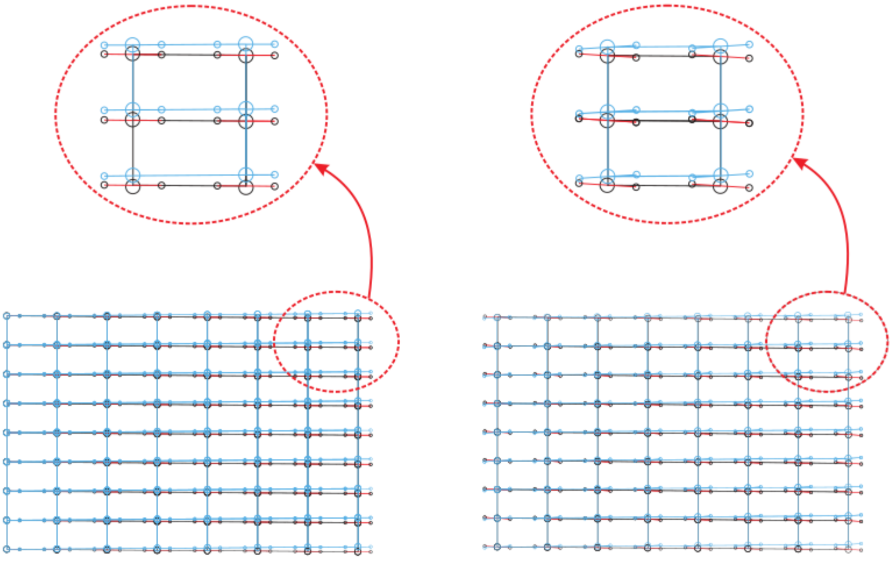

Fig. S3. The weak monoclinic distortion evidenced by high-resolution ESRF data ($\sim$0.4° in the α angle), combined with the presence of two twin domains related by a twofold axis parallel to b and a modulation vector $\mathbf{q}$ = 0.25 b^*, allows the generation of a diffraction pattern in which a splitting that increases with k is observed for both main and satellite reflections. However, the asymmetric splitting observed in the experimental data (see Fig. 3) between the +1 and - 1 satellites of the same main reflection cannot be reproduced by this model. To account for this effect, a small component along c^* must be added to the modulation vector; this component is estimated to be - 0.019 c^*. The resulting very long periodicity appears to be intrinsic to the CDW.

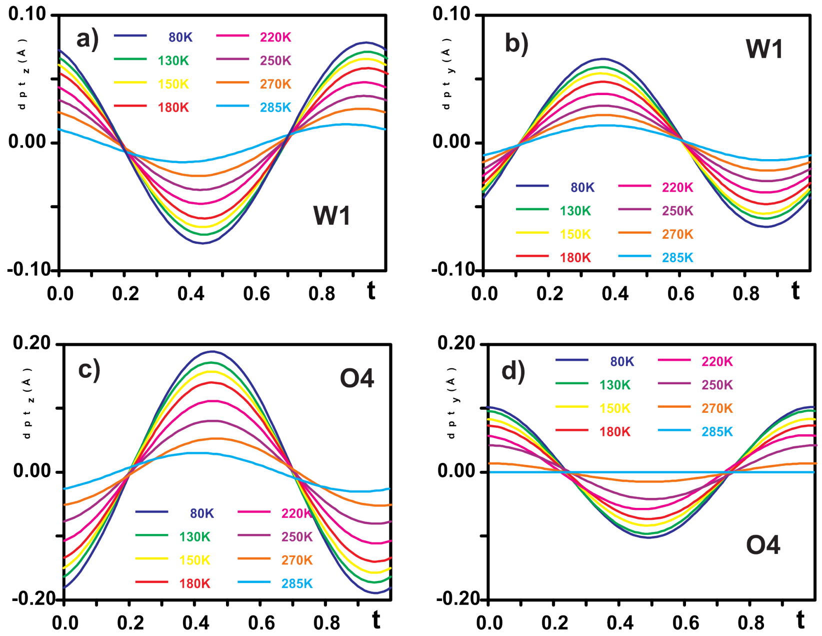

Fig. S4. Evolution versus t of the atomic displacements for W1 and O4 for different temperatures.

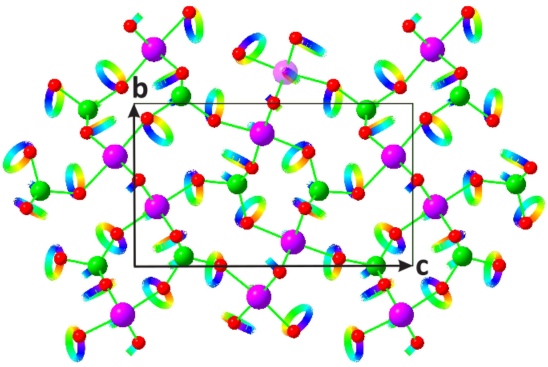

Fig. S5. Projection along **a** of $P_4W_4O_{20}$ at 80K exhibiting for each atoms the Lissajous traces of the atomic displacements due to the modulation. The displacements have been magnified 2.5 times. Events occurring at the same t have the same colors in the traces. Molecoolqt (**?**) has been used to create this figure.

Fig. S6 shows the zoom of the 0KL and H0.75L maps around the satellite 0,0.75,$\bar{5}$.

We can see the dependence of the diffuse scattering on the temperature and the strong

intensity of the new satellite reflection at the CDW ground state. For the IXS measurement, the temperature of transition was checked through an elastic measurement of the rocking curve of the Bragg peak 0,0.75,$\bar{6}$, Fig. S7 and its fitting was rather complicated. The transition should be reached, when the FWHM is just the resolution, so the order parameter converge to infinite. This temperature is obtained with the crossing of the linear fitting of the Lorentzian FWHM at y=0.2, which is more or less the resolution of the instrument (or the broadening of this peak in particular), which gives 280K, as shown in Fig. S8.

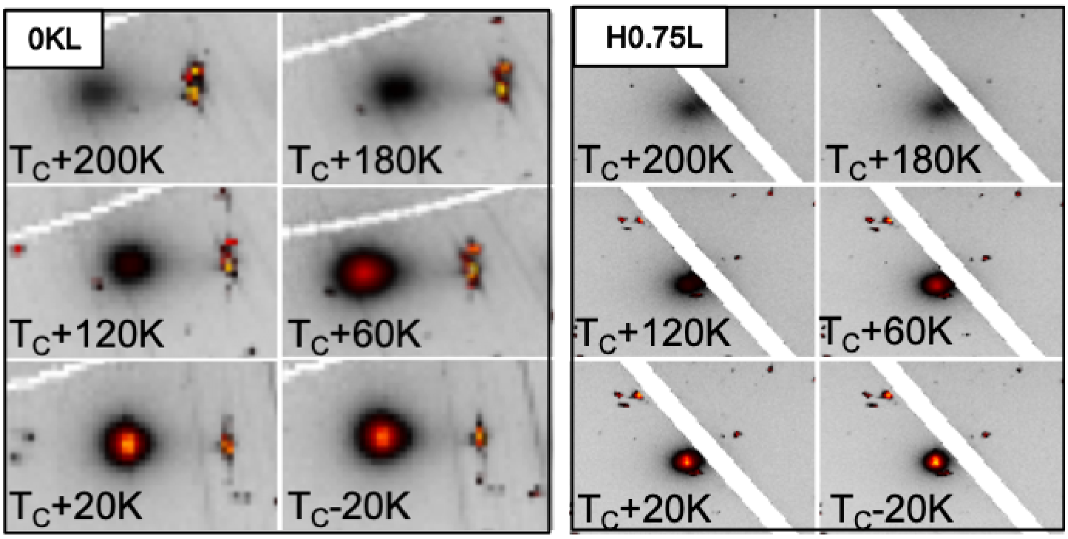

Fig. S6. 0KL and H0.75L maps at different temperatures, around the satellite position at 0,0.75,$\bar{5}$.

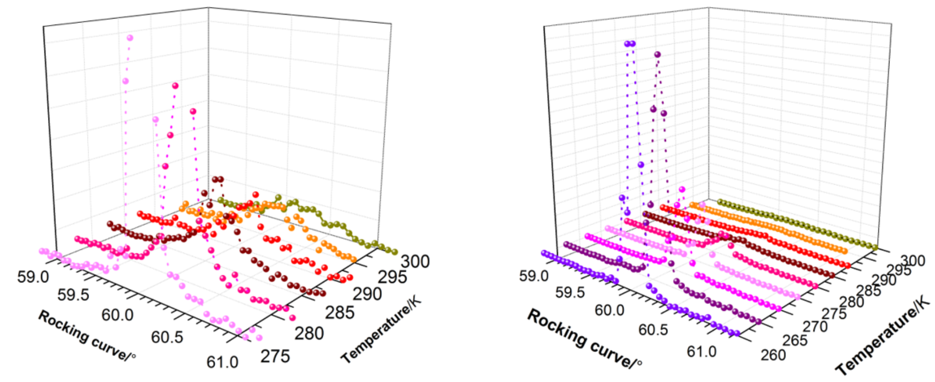

Fig. S7. Temperature dependence of the rocking curve for the satellite of m=2 made on the spectrometer on the CDW satellite, $\mathbf{q}$=∼-0.25$\mathbf{b^*}$, in the point Q(0 0.75 -6), from 300 K to 275 K (on the left), and until the transition (on the right), where the intensity increase and the Bragg peak is formed.

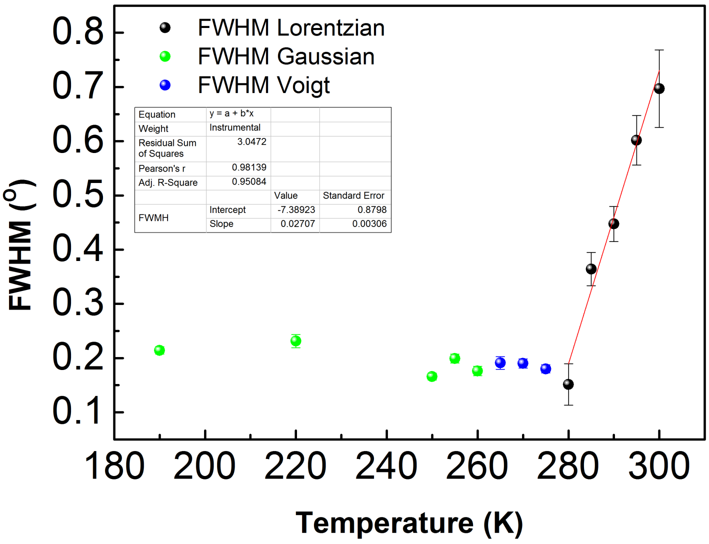

Fig. S8. Full Width Half Maxima (FWHM) of the Pseudo-Voigt, Gaussian and Lorentzian fitting are reported for the IXS scans at different temperatures.

Appendix 3
Additional tables

Table S1 summarized the information on data collections and refinement at RT and 80K. Tables (S2,S3) and (S4,S5) provide the structural parameters refined at RT and 80K respectively. Table S6 offers a quantification of the effect of the modulation at 80K on the distortion of the polyhedra.

	RT	80K
chemical formula	$P_4W_4O_{20}$	$P_4W_4O_{20}$
Space group	Pmcn	
$P2_1/m$ **a** (Å)	5.22786(5)	5.2302(2)
**b** (Å)	6.55814(11)	6.5427(5)
**c** (Å)	11.1883(2)	11.1823(4)
α (°)	90.0583(15)	89.900(5)
β (°)	90.0118(12)	89.984(3)
γ (°)	90.0024(10)	89.979(5)
Volume (Å^3)	383.591(12)	382.65(4)
q wave vector	-	0.2502(11) $\vec{b}^\star$
Z	1	
h limit	$-8 \leq h \leq 8$	$-8 \leq h \leq 8$
k limit	$-11 \leq k \leq 10$	$-11 \leq k \leq 11$
l limit	$-18 \leq l \leq 18$	$-17 \leq l \leq 18$
reflections $[I \geq 3\sigma(I)]$	952	2254
main	952	911
1st order satellite	-	1343
Rint (%)	0.05	
no of refinement parameters	41	48
R_F (%)	2.08	3.49
$R_{F,0}$ (%)	-	2.45
$R_{F,1}$ (%)	-	6.23

Table S1. *Refinement parameters*

atom	occupancy	x	y	z	u_{iso} ($\AA^2$)
W1	1	0.25	-0.16856(2)	0.438765(13)	0.00569(6)
P1	1	0.25	0.47108(16)	0.65538(10)	0.0067(2)
O1	1	0.25	0.6695(5)	0.5859(4)	0.0248(14)
O2	1	0.25	0.5209(6)	0.7871(3)	0.0188(10)
O3	1	0.0124(9)	0.3535(7)	0.6263(4)	0.0560(15)
O4	1	0	1	0.5	0.0283(12)

Table S2. *Positional parameters at RT*

atom	u_{11}	u_{22}	u_{33}
W1	0.00406(11)	0.00787(11)	0.00514(11)
P1	0.0073(4)	0.0081(4)	0.0046(4)
O1	0.043(3)	0.0158(19)	0.0150(18)
O2	0.029(2)	0.0235(17)	0.0041(14)
O3	0.069(3)	0.077(3)	0.0227(19)
O4	0.031(2)	0.044(3)	0.0098(16)
atom	u_{12}	u_{13}	u_{23}
W1	0	0	-0.00048(4)
P1	0	0	-0.0002(3)
O1	0	0	0.0100(12)
O2	0	0	-0.0051(11)
O3	-0.065(2)	-0.0160(18)	0.0060(16)
O4	0.0271(19)	0.0098(15)	0.0107(15)

Table S3. *ADP harmonic parameters (Å^2) at RT*

atom	occupancy	harmonic	$\langle x \rangle$	$\langle y \rangle$	$\langle z \rangle$	u_{iso} (Å^2)
W1	1		0.25	-0.16828(3)	0.438710(15)	0.00414(7)
		s,1	0	0.0091	-0.004220(18)	
		c,1	0	-0.0043	0.005616(19)	
P1	1		0.25	0.47061(19)	0.65512(11)	0.0055(3)
		s,1	0	0.0092	-0.00727(12)	
		c,1	0	0.0046	-0.00442(12)	
O1	1		0.25	0.5213(5)	0.7872(3)	0.0124(10)
		s,1	0	0.0066	-0.0073(3)	
		c,1	0	-0.0296	0.0005(4)	
O2	1		0.25	0.6673(6)	0.5837(4)	0.0192(13)
		s,1	0	0.0153	-0.0035(4)	
		c,1	0	0.0205	0.0141(4)	
O3	1		0	1	0.5	0.0287(15)
		s,1	0.0003(9)	0.0129(8)	-0.0072(4)	
		c,1	0	0	0	
O4	1		0.0116(10)	0.3515(7)	0.6270(3)	0.0536(17)
		s,1	-0.0003(9)	0.0078(7)	-0.0043(3)	
		c,1	0.0004(9)	0.0136(8)	-0.0163(4)	

Table S4. *Positional parameters at 80K. Atomic displacement along x,y,z are described by u = s,1 sin 2 π* $\overline{x}_4 + c,1cos2\pi\ \overline{x}_4$.

atom	u_{11}	u_{22}	u_{33}
W1	0.00276(11)	0.00724(11)	0.00242(11)
P1	0.0064(5)	0.0075(5)	0.0026(5)
O1	0.0215(19)	0.0138(15)	0.0018(14)
O2	0.039(3)	0.0126(17)	0.0064(16)
O3	0.030(3)	0.049(3)	0.0070(18)
O4	0.072(3)	0.075(3)	0.0138(17)
atom	u_{12}	u_{13}	u_{23}
W1	0	0	-0.00009
P1	0	0	-0.000393
O1	0	0	-0.001928
O2	0	0	0.003109
O3	0.029(2)	0.0089(18)	0.0094(19)
O4	-0.067(3)	-0.0148(18)	0.0086(18)

Table S5. *ADP harmonic parameters at 80K*

Polyhedron	Distances (Å)	Angles (°)
WO_6	$d_{W1-O1^{(1)}} = 1.955(5) \pm 0.011$<br>$d_{W1-O2^{(2)}} = 1.952(5) \pm 0.011$<br>$d_{W1-O3^{(2)}} = 1.843(3) \pm 0.003$<br>$d_{W1-O3^{(3)}} = 1.843(3) \pm 0.003$<br>$d_{W1-O4^{(3)}} = 1.964(7) \pm 0.006$<br>$d_{W1-O4^{(4)}} = 1.964(7) \pm 0.006$	$O1^{(1)}-\widehat{W1}-O2^{(2)} = 176.00(13) \pm 1.00$<br>$O1^{(1)}-\widehat{W1}-O3^{(2)} = 91.66(15) \pm 0.93$<br>$O1^{(1)}-\widehat{W1}-O3^{(3)} = 91.66(15) \pm 0.93$<br>$O1^{(1)}-\widehat{W1}-O4^{(5)} = 88.62(17) \pm 0.52$<br>$O1^{(1)}-\widehat{W1}-O4^{(4)} = 88.62(17) \pm 0.52$<br>$O2^{(2)}-\widehat{W1}-O3^{(2)} = 91.18(16) \pm 0.74$<br>$O2^{(2)}-\widehat{W1}-O3^{(3)} = 91.18(16) \pm 0.74$<br>$O2^{(2)}-\widehat{W1}-O4^{(5)} = 88.48(18) \pm 0.74$<br>$O2^{(2)}-\widehat{W1}-O4^{(4)} = 88.48(18) \pm 0.74$<br>$O3^{(2)}-\widehat{W1}-O3^{(3)} = 90.43(18) \pm 0.23$<br>$O3^{(2)}-\widehat{W1}-O4^{(5)} = 178.60(2) \pm 0.40$<br>$O3^{(2)}-\widehat{W1}-O4^{(4)} = 90.7(2) \pm 0.3$<br>$O3^{(3)}-\widehat{W1}-O4^{(5)} = 90.7(2) \pm 0.3$<br>$O3^{(3)}-\widehat{W1}-O4^{(4)} = 178.60(2) \pm 0.40$<br>$O4^{(5)}-\widehat{W1}-O4^{(4)} = 88.3(3) \pm 0.4$
PO_4	$d_{P1-O1^{(0)}} = 1.522(6) \pm 0.009$<br>$d_{P1-O2^{(0)}} = 1.527(3) \pm 0.016$<br>$d_{P1-O4^{(0)}} = 1.506(7) \pm 0.005$<br>$d_{P1-O4^{(6)}} = 1.506(7) \pm 0.005$	$O1^{(0)}-\widehat{P1}-O2^{(0)} = 109.2(2) \pm 0.3$<br>$O1^{(0)}-\widehat{P1}-O4^{(0)} = 108.6(2) \pm 0.3$<br>$O1^{(0)}-\widehat{P1}-O4^{(7)} = 108.6(2) \pm 0.3$<br>$O2^{(0)}-\widehat{P1}-O4^{(0)} = 109.3(2) \pm 0.3$<br>$O2^{(0)}-\widehat{P1}-O4^{(7)} = 109.3(2) \pm 0.3$<br>$O4^{(0)}-\widehat{P1}-O4^{(7)} = 111.9(4) \pm 0.7$

Table S6. *Quantification of polyhedra distortion due to the modulation at 80 K (average value ± deviation due to the modulation). Symmetry codes: (0) x, y, z; (1) $-x+12, -y+12, z-12$; (2) $x, y-1, z$; (3) $x+12, -y+1, -z+1$; (4) $-x, -y, -z+1$; (5) $x+12, -y, -z+1$; (6) $x+12, y, z$; (7) $-x+12, y, z$.*
